# Author Correction: High-density information storage in an absolutely defined aperiodic sequence of monodisperse copolyester

**DOI:** 10.1038/s41467-021-25676-3

**Published:** 2021-09-03

**Authors:** Jung Min Lee, Mo Beom Koo, Seul Woo Lee, Heelim Lee, Junho Kwon, Yul Hui Shim, So Youn Kim, Kyoung Taek Kim

**Affiliations:** 1 Department of Chemistry, Seoul National University, Seoul, 08826 Korea; 2 Department of Chemical Engineering, Ulsan National Institute of Science and Technology (UNIST), Ulsan, 44919 Korea

**Keywords:** Polymers, Polymer synthesis

Correction to: *Nature Communications* 10.1038/s41467-019-13952-2, published online 07 January 2020.

The original version of the Supplementary Information associated with this Article contained several errors on pages 6 and 7, which incorrectly rounded the calculated mass of five tetramers to even numbers. Additionally, there was a typo in the legend for Supplementary Table 15 on page 56. The correct version can be found as Supplementary Information associated with this Correction. The errors have been corrected in the Supplementary Information associated with this Article.

## Supplementary information

Supplementary Information

