# Supplementary material for: High-density information storage in an absolutely defined aperiodic sequence of monodisperse copolyester
Source: Nat Commun. 2020 Jan 7;11:56. doi: 10.1038/s41467-019-13952-2 (PMC6946701; doi:10.1038/s41467-019-13952-2)
Supplement: Supplementary file 1 — Supplementary Information [file 41467_2019_13952_MOESM1_ESM.pdf]

Supplementary Information

for

High-density information storage in an absolutely defined aperiodic sequence of monodisperse copolyester

Jung Min Lee[1], Mo Beom Koo[1], Seul Woo Lee[1], Heelim Lee[1], Junho Kwon[1], Yul Hui Shim[2], So Youn Kim[2] and Kyoung Taek Kim[1,*]

[1]Department of Chemistry, Seoul National University, Seoul 08826, Korea

[2]Department of Chemical Engineering, Ulsan National Institute of Science and Technology (UNIST), Ulsan 44919, Korea

*Correspondence to: ktkim72@snu.ac.kr

Supplementary Methods

Materials

rac-lactide (Alfa Aesar) was recrystallized from MeOH/EtOAc mixture prior to use. *rac*-phenyllactic acid (AKSci) was used without further purification. *rac*-Lactic acid, ethyl lactate, benzyl chloride (99%), triethylamine (≥99%), imidazole (99%), *tert*-butyldimethylsilyl chloride (97%), boron trifluoride diethyl etherate, palladium on activated charcoal (10%) and 4-(dimethylamino)pyridine (DMAP) were purchased from Sigma Aldrich and used without further purification. 1-(3-Dimethylaminopropyl)-3-ethylcarbodiimide hydrochloride (EDC•HCl) was purchased from Tokyo Chemical Industry and used without purification. Dichloromethane (DCM), dimethylformamide (DMF), and toluene were distilled over CaH_2 under N_2.

Characterization

^{1}H NMR spectra were recorded on a Varian 500 MHz using $CDCl_3$ as solvent. ^{13}C NMR spectra were recorded on an Agilent 400-MR DD2 Magnetic Resonance System using $CDCl_3$ as solvent. Gel permeation chromatography (GPC) was performed on an Agilent 1260 Infinity equipped with a PL gel 5 μm mixed D column and differential refractive index detectors. THF was used as an eluent with a flow rate of 0.3 mL min^{-1} at 35 °C. A polystyrene standard kit (Agilent Technologies) was used for calibration. Automated column chromatography was performed on a Biotage SP1 flash chromatography purification system equipped with a silica column cartridge (KP-Sil 100g and 50g). n-hexane and ethyl acetate were used as eluent. Differential Scanning Calorimetry (DSC) was performed on a TA Instruments Q10 from –40 °C to 140 °C with a scan rate of 10 °C min^{-1} under N_2 atmosphere. The instrument was calibrated before measurements by measuring the melting temperature of indium (T_m = 429.75 K). Mass spectra of PAHs were measured on a Bruker Ultraflex III TOF-TOF mass spectrometer equipped with a Nd:YAG laser (355 nm). The instrument was operated in a positive ion mode. External calibration was conducted using the following peptides and proteins as reference: Bradykinin fragment 1-7 (757.3997 Da), Angiotensin II (1046.5423 Da), $P_{14}R$ (1533.8582 Da), ACTH fragment 18-39 (2465.1989 Da), Insulin oxidized B chain (3494.6513 Da), Insulin (5735 Da), Cytochrome c (12362 Da), Apomyoglobin (16952 Da), Aldolase (39212 Da) and Albumin (66430 Da)). The ion is $[M+H]^+$ ion. 2-(4-Hydroxyphenylazo)benzoic acid (HABA) or *trans*-2-[3-(4-*tert*-Butylphenyl)-2-methyl-2-propenylidene]malononitrile (DCTB) was used as a matrix.

General procedure for the convergent growth of poly(α-hydroxy acid)s (PAHs)

General procedure for deprotection of the benzyl group by hydrogenation

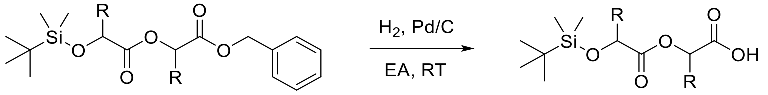

A compound protected with *tert*-butyldimethylsilyl (TBDMS) and benzyl groups was dissolved in ethyl acetate. Palladium on activated charcoal (10% Pd/C, 0.03~0.2 eq.) was added to the solution, and the suspension was purged with argon for 15 minutes. The argon atmosphere was then replaced with hydrogen atmosphere, and the reaction mixture was stirred at room temperature. The reaction was monitored by thin layer chromatography (TLC) analysis. Upon completion of the reaction, the suspension was filtered through a Celite cake to remove Pd/C. The product was obtained by removing the solvent from the filtrates under reduced pressure.

General procedure for deprotection of the TBDMS group with fluoride

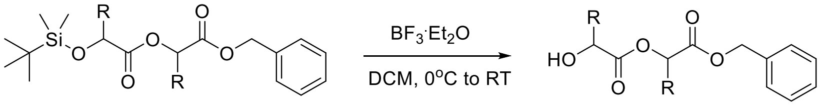

A compound protected with TBDMS and benzyl groups was dissolved in dry dichloromethane (DCM). The solution was cooled to 0°C on an ice bath, and boron trifluoride diethyl etherate (BF_3•Et_2O) was added dropwise. The reaction mixture was stirred at room temperature for 4 h. The reaction was monitored by TLC analysis. Upon completion of the reaction, the reaction was quenched with saturated $NaHCO_3$ followed by dilution with water. The organic layer was separated and washed with brine. The combined organic layer was dried over $MgSO_4$, and the solvent was removed under reduced pressure. The crude product was purified by automated column chromatography.

General procedure for esterification reactions

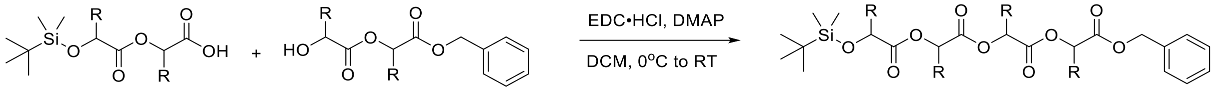

Alcohol and carboxylic acid (1.05 eq.) were dissolved in dry DCM, and the mixture was cooled to 0°C on an ice bath. To the mixture, 4-(dimethylamino)pyridine (DMAP, 0.2 eq.) and 1-(3-dimethylaminopropyl)-3-ethylcarbodiimide hydrochloride (EDC•HCl, 1.4 eq.) were added. The reaction mixture was stirred overnight at room temperature, and the reaction was monitored by TLC analysis. Upon completion of the reaction, the reaction mixture was washed with water and brine. The combined organic layer was dried over $MgSO_4$, and the solvent was removed under reduced pressure. The crude product was purified by automated column chromatography. Products with 32 repeating units or more were purified by preparative-size exclusion chromatography (prep-SEC) with a series of columns using chloroform as an eluent.

Synthesis of the constituent units of PAHs

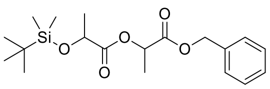

LA2

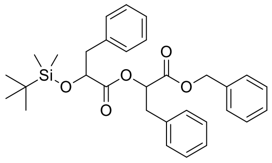

PA2

LA2 was synthesized according to the literature (Refs. 32 and 33 in the main text). Colorless liquid (25 g, 91%); ^{1}H NMR (500MHz, $CDCl_3$): δ 7.37-7.27 (m, 5H, Ph-**H**), 5.18 (m, 3H, COO-C**H**(CH_3)C=O and Ph-C**H$_2$**-O), 4.39 (q, *J* = 6.8Hz, 1H, SiO-C**H**(CH_3)C=O), 1.53 (d, *J* = 7.1 Hz, 3H, C**H$_3$**-CH), 1.43 (d, *J* = 6.8 Hz, 1H, C**H$_3$**-CH), 0.88 (s, 9H, (C**H$_3$**)$_3$C-Si), 0.10 (dd, *J* = 12.2, 5.7 Hz, 6H, (CH_3)$_3$C-Si(C**H$_3$**)$_2$-O) ppm; ^{13}C NMR (400MHz, $CDCl_3$): δ 173.2 (SiO-CH(CH_3)**C**=O), 170.1 (COO-CH(CH_3)**C**=O), 135.3 (**C$_5$**H$_5$-CH$_2$-COO), 128.5 (**C$_5$**H$_5$-CH$_2$-COO), 128.3 (**C$_5$**H$_5$-CH$_2$-COO), 128.1 (**C$_5$**H$_5$-CH$_2$-COO), 68.7 (CH_3-**C**H-SiO), 68.0 (CH_3-**C**H-COO), 66.8 (C_5H_5-**C**H$_2$-COO), 25.7 ((CH_3)$_3$**C**-Si(CH_3)$_2$-O), 21.1 ((**C**H$_3$)$_3$C-Si(CH_3)$_2$-O), 18.2 (**C**H$_3$-CH-SiO), 16.8 (**C**H$_3$-CH-COO), -4.9 ((CH_3)$_3$C-Si(**C**H$_3$)$_2$-O), -5.3 ((CH_3)$_3$C-Si(**C**H$_3$)$_2$-O) ppm; MS (ESI/Q-TOF): m/z (isotopic maximum value) calcd for $C_{19}H_{30}O_5Si+Na^+$ [**M**+Na]$^+$: 389; found 390.

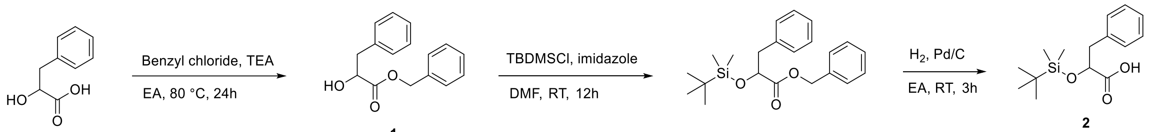

1 2

Benzyl 2-hydroxy-3-phenylpropanoate (HO-PA-Bz) (**1**). A two-neck round bottom flask (RBF) was charged with *rac*-phenyllactic acid (20 g, 120.34 mmol), triethylamine (TEA, 144.42 mmol), benzyl chloride (132.39 mmol), and ethyl acetate (120 mL). The reaction flask was connected to a condenser and was refluxed for 24 h. Upon completion of the reaction, the reaction mixture was washed with water and brine. The combined organic layer was dried over $MgSO_4$, and the solvent was removed under reduced pressure. The crude mixture was purified by column chromatography using n-hexane/EA (4:1 v/v) as an eluent. The product **1** was obtained as yellow liquid (27.39 g, 89%); ^{1}H NMR (500MHz, $CDCl_3$): 7.43-7.12 (m, 10H, Ph-**H**), 5.19 (m, 2H, Ph-C**H$_2$**-O), 4.50 (d, *J* = 4.50 Hz, 1H, HO-C**H**(CH_2Ph)C=O), 3.13 (dd, *J* = 13.9, 4.6 Hz, 1H, Ph-C**H$_2$**-CH), 2.98 (dd, *J* = 13.9, 6.5 Hz, 1H, Ph-C**H$_2$**-CH), 2.72 (b, 1H, O**H**) ppm.

2-((*tert*-butyldimethylsilyl)oxy)-3-Phenylpropanoic acid (TBDMS-PA-COOH) (**2**). **1** (12g, 46.82 mmol) was dissolved in dry DMF (50 mL). To this solution, imidazole (70 mmol) and *tert*-butyldimethylchlorosilane (TBDMS-Cl, 56 mmol) were added. The reaction mixture was stirred at room temperature for 12 h. Upon completion of the reaction, the reaction was quenched with saturated $NaHCO_3$ (20 mL), followed by dilution with water (40 mL). The resulting mixture was extracted with n-hexane. The combined organic layer was washed with brine and dried over $MgSO_4$, and the solvent was removed under reduced pressure. The crude mixture was purified by column chromatography using n-hexane/EA (24:1 v/v) as an eluent. Benzyl 2-((tert-butyldimethylsilyl)oxy)-3-phenylpropanoate (TBDMS-PA-Bz) was obtained as colorless oil (15.84 g, 91%). TBDMS-PA-Bz was subsequently hydrogenated to yield the title compound **2** (12 g, quant.). TBDMS-PA-Bz; ^{1}H NMR (500MHz, $CDCl_3$): 7.40-7.12 (m, 10H, Ph-**H**), 5.16 (q, *J* = 12.2Hz, 2H, Ph-C**H$_2$**-O), 4.38 (dd, *J* = 8.9, 4.1 Hz, 1H, SiO-C**H**(CH_2Ph)C=O), 3.09 (dd, *J* = 8.9, 4.1 Hz, 1H, Ph-C**H$_2$**-CH), 2.91 (dd, *J* = 13.4, 8.9 Hz, 1H, Ph-C**H$_2$**-CH), 0.77 (s, *J* = 2.8 Hz, 9H, (C**H$_3$**)$_3$C-Si), -0.19 (d, *J* = 46.2 Hz, 6H, (CH_3)$_3$C-Si(C**H$_3$**)$_2$-O) ppm. TBDMS-PA-COOH (**2**); ^{1}H NMR (500MHz, $CDCl_3$): 7.30-7.15 (m, 5H, Ph-**H**), 4.40 (dd, *J* = 8.1, 3.7 Hz, 1H, SiO-C**H**(CH_2Ph)C=O), 3.11 (dd, *J* = 13.7, 3.7 Hz, 1H, Ph-C**H$_2$**-CH), 2.91 (dd, *J* = 13.4, 8.9 Hz, 1H, Ph-C**H$_2$**-CH), 0.83 (s, 9H, (C**H$_3$**)$_3$C-Si), -0.15 (d, *J* = 46.2 Hz, 6H, (CH_3)$_3$C-Si(C**H$_3$**)$_2$-O) ppm.

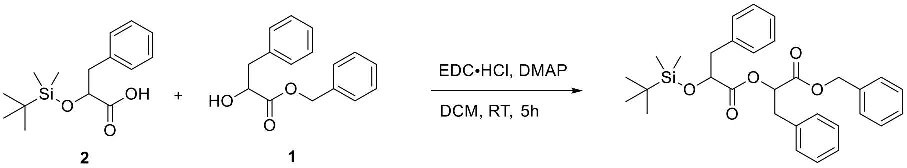

2 1

PA2

PA2. The equimolar mixture of **1** (11g, 42.92 mmol) and **2** (12g, 42.79 mmol) was dissolved in dry DCM, and the solution was cooled to 0°C on an ice-bath. DMAP (8.56 mmol) and EDC•HCl (55.63 mmol) were then introduced to the cooled solution. The reaction mixture was stirred at 0°C for 1 h and at room temperature for 5 h. Upon completion of the reaction, the reaction mixture was concentrated under reduced pressure, and the crude mixture was subjected to automated column chromatography using n-hexane/EA

(19:1 v/v) as an eluent. The product **PA2** was obtained as colorless oil (16.07 g, 72%); ^{1}H NMR (500MHz, $CDCl_3$): 7.37-7.01 (m, 15H, Ph-**H**), 5.32 (m, 1H, COO-C**H**(CH_3)C=O), 5.14 (m, 2H, Ph-C$\mathbf{H_2}$-O), 4.30 (ddd, *J* = 29.2, 9.2, 3.4 Hz, 1H, SiO-C**H**(CH_2Ph)C=O), 2.90 (m, 4H, Ph-C$\mathbf{H_2}$-CH), 0.71 (d, *J* = 91. Hz, 9H, (C$\mathbf{H_3}$)$_3$C-Si), -0.29 (dd, *J* = 51.5, 23.9 Hz, 6H, (CH_3)$_3$C-Si(C$\mathbf{H_3}$)$_2$-O) ppm; ^{13}C NMR (400MHz, $CDCl_3$): δ 172.4 (SiO-CH(CH_2Ph)**C**=O), 169.0 (COO-CH(CH_2Ph)**C**=O), 137.7 ($\mathbf{C_5}H_5$-CH_2-COO), 135.8 ($\mathbf{C_5}H_5$-CH_2-CH-SiO), 135.4 ($\mathbf{C_5}H_5$-CH_2-CH-COO), 130.0 ($\mathbf{C_5}H_5$-CH_2-CH-SiO, $\mathbf{C_5}H_5$-CH_2-CH-COO and $\mathbf{C_5}H_5$-CH_2-COO), 128.7 ($\mathbf{C_5}H_5$-CH_2-CH-SiO, $\mathbf{C_5}H_5$-CH_2-CH-COO and $\mathbf{C_5}H_5$-CH_2-COO), 127.2 ($\mathbf{C_5}H_5$-CH_2-CH-SiO, $\mathbf{C_5}H_5$-CH_2-CH-COO and $\mathbf{C_5}H_5$-CH_2-COO), 73.5 (Ph-CH_2-**C**H-SiO), 73.2 (Ph-CH_2-**C**H-COO), 67.2 (C_5H_5-**C**H_2-COO), 41.6 (Ph-**C**H_2-CH-SiO), 37.4 (Ph-**C**H_2-CH-COO), 25.8 ((**C**H_3)$_3$C-Si(CH_3)$_2$-O), 18.3 ((CH_3)$_3$**C**-Si(CH_3)$_2$-O), -5.2 ((CH_3)$_3$C-Si(**C**H_3)$_2$-O), -5.7 ((CH_3)$_3$C-Si(**C**H_3)$_2$-O) ppm; MS (ESI/Q-TOF): m/z calcd for $C_{31}H_{38}O_5Si+Na^+$ [**M**+Na]$^+$: 541; found 542.

Synthesis of poly(phenyllactic-*co*-lactic acid) (PcL)

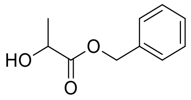

HO-LA-Bz

Benzyl 2-hydroxypropanoate (HO-LA-Bz) was synthesized as previously described in the synthesis of HO-PA-Bz. Colorless liquid (20 g, 82%); ^{1}H NMR (500MHz, $CDCl_3$): δ 7.52-7.24 (m, 5H, Ph-**H**), 5.21 (s, 2H, Ph-C$\mathbf{H_2}$-O), 4.32 (q, *J* = 6.9 Hz, 1H, HO-C**H**(CH_3)C=O), 2.80 (b, 1H, O**H**), 1.44 (d, *J* =6.9 Hz, 1H, $\mathbf{CH_3}$-CH) ppm.

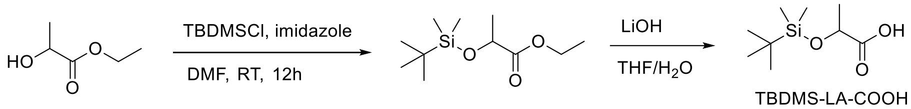

TBDMS-LA-COOH

TBDMS-LA-COOH was synthesized in two steps according to the literature with slight modifications (Ref. 42 in the main text). Ethyl lactate (4 g, 34.88 mmol) was dissolved in dry DMF (35 mL). To this solution, imidazole (52 mmol) and TBDMSCl (41 mmol) were added. The reaction mixture was stirred at room temperature for 12 h. Upon completion of the reaction, the reaction was quenched with saturated $NaHCO_3$ (15 mL), followed by dilution with water (30 mL). The resulting mixture was extracted with n-hexane. The combined organic layer was washed with brine and dried over $MgSO_4$, and the solvent was removed under reduced pressure. Colorless liquid (6.42 g, 79%); ^{1}H NMR (500MHz, $CDCl_3$): δ 4.29 (q, *J* = 6.8 Hz, 1H, O-C**H**(CH_3)C=O), δ 4.16 (m, 2H, CH_3-C$\mathbf{H_2}$-O), 1.38 (d, *J* = 6.8 Hz, 3H, C$\mathbf{H_3}$-CH), 1.26 (t, *J* = 7.1 Hz, C$\mathbf{H_3}$-CH_2), 0.89 (s, 9H, (C$\mathbf{H_3}$)$_3$C-Si), 0.07 (d, *J* = 11.7 Hz, 6H, (CH_3)$_3$C-Si(C$\mathbf{H_3}$)$_2$-O) ppm.

Subsequently, the product (6 g, 25.82 mmol) was dissolved in dry THF (280 ml) and was cooled to 0°C on an ice bath. An aqueous LiOH solution (1.2M, 100 mL) was added dropwise over the next 15 minutes. The ice bath was removed and the reaction was stirred for 4 h. When the reaction was complete, water was added to the reaction flask and the solvent was removed under reduced pressure. The resulting mixture was extracted with Et_2O to remove the starting material. The organic layers were discarded. The combined aqueous layer was then acidified to pH 3~4 using 1.0 M HCl, then was extracted with Et_2O. The combined organic phase was dried over MgSO4 and was concentrated under reduced pressure to give the title compound. A white solid (2.71 g, 51 %); ^{1}H NMR (500MHz, $CDCl_3$): δ 4.36 (q, *J* = 6.8 Hz, 1H, O-C**H**(CH_3)C=O), 1.45 (d, *J* = 6.8 Hz, 3H, C$\mathbf{H_3}$-CH), 0.92 (m, 9H, (C$\mathbf{H_3}$)$_3$C-Si), 0.12 (d, *J* = 23.2 Hz, 6H, (CH_3)$_3$C-Si(C$\mathbf{H_3}$)$_2$-O) ppm.

LA2 and **PA2** were used as **LL** and **PP**.

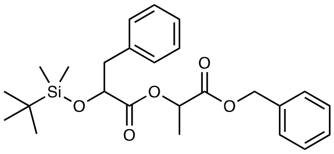

PL

PL. Colorless liquid (20.21 g, 74%); ^{1}H NMR (500MHz, $CDCl_3$): δ 7.43-6.91 (m, 10H, Ph-**H**), 5.15 (m, 3H, COO-C**H**(CH_3)C=O and Ph-C**H$_2$**-O), 4.36 (td, *J* = 9.5, 3.5 Hz, 1H, SiO-C**H**(CH_2Ph)C=O), 3.17-2.76 (m, 2H, Ph-C**H$_2$**-CH), 1.49 (m, 3H, C**H$_3$**-CH), 0.75 (m, 9H, (C**H$_3$**)$_3$C-Si), -0.21 (m, 6H, $(CH_3)_3$C-Si(C**H$_3$**)$_2$-O) ppm; MS (ESI/Q-TOF): m/z (isotopic maximum value) calcd for $C_{25}H_{34}O_5Si+Na^+$ [**M**+Na]$^+$: 465; found 466.

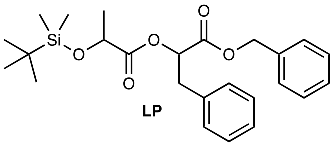

LP

LP. Colorless liquid (2.73g, 71%); ^{1}H NMR (500MHz, $CDCl_3$): δ 7.46-6.96 (m, 10H, Ph-**H**), 5.35 (m, 1H, COO-C**H**(CH_2Ph)C=O), 5.15 (m, 2H, Ph-C**H$_2$**-O), 4.34 (m, 1H, SiO-C**H**(CH_3)C=O), 3.27-3.08 (m, 2H, Ph-C**H$_2$**-CH), 1.31 (dd, *J* = 47.3, 6.8 Hz, 3H, C**H$_3$**-CH), 0.88 (dd, *J* = 6.9, 4.9 Hz, 9H, (C**H$_3$**)$_3$C-Si), 0.02 (dd, *J* = 15.6, 13.5 Hz, 6H, $(CH_3)_3$C-Si(C**H$_3$**)$_2$-O) ppm; MS (ESI/Q-TOF): m/z calcd for $C_{25}H_{34}O_5Si+Na^+$ [**M**+Na]$^+$: 465; found 466.

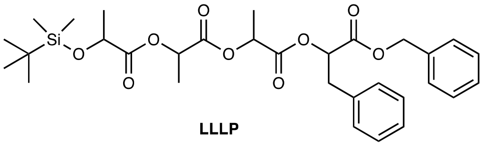

LLLP

LLLP. Colorless oil (2.78 g, 93%); ^{1}H NMR (500MHz, $CDCl_3$): δ 7.37-7.10 (m, 10H, Ph-**H**), 5.34 (dd, *J* = 6.9, 5.3 Hz, 1H, COO-C**H**(CH_2Ph)C=O), 5.25-5.03 (m, 4H, COO-C**H**(CH_3)C=O and Ph-C**H$_2$**-O), 4.41 (q, *J* = 6.7 Hz, 1H, SiO-C**H**(CH_3)C=O), 3.16 (m, 2H, Ph-C**H$_2$**-CH), 1.57-1.32 (m, 9H, C**H$_3$**-CH), 0.92 (s, 9H, (C**H$_3$**)$_3$C-Si), 0.10 (dd, *J* = 9.9, 3.4 Hz, 6H, $(CH_3)_3$C-Si(C**H$_3$**)$_2$-O) ppm; MS (ESI/Q-TOF): m/z calcd for $C_{31}H_{42}O_9Si+Na^+$ [**M**+Na]$^+$: 609; found 610.

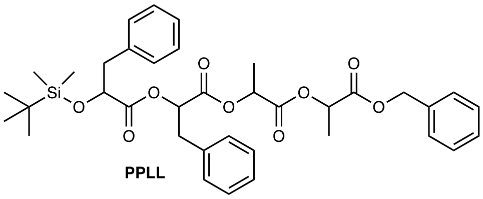

PPLL

PPLL. Colorless oil (5.19 g, 97%); ^{1}H NMR (500MHz, $CDCl_3$): δ 7.40-7.00 (m, 15H, Ph-**H**), 5.36 (m, 1H, COO-C**H**(CH_2Ph)C=O), 5.30-5.12 (m, 4H, COO-C**H**(CH_3)C=O and Ph-C**H$_2$**-O), 4.32 (m, 1H, SiO-C**H**(CH_2Ph)C=O), 3.45-2.55 (m, 4H, Ph-C**H$_2$**-CH), 1.59-1.42 (m, 6H, C**H$_3$**-CH), 0.74 (m, 9H, (C**H$_3$**)$_3$C-Si), -0.26 (dd, 6H, $(CH_3)_3$C-Si(C**H$_3$**)$_2$-O) ppm; MS (ESI/Q-TOF): m/z calcd for $C_{37}H_{46}O_9Si+Na^+$ [**M**+Na]$^+$: 685; found 686.

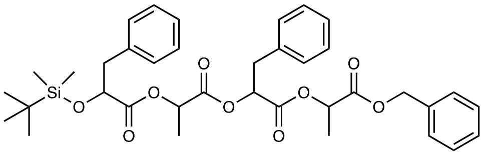

PLPL

PLPL. Colorless oil (12.51 g, 92%); ^{1}H NMR (500MHz, $CDCl_3$): δ 7.40-7.20 (m, 15H, Ph-**H**), 5.36 (m, 1H, COO-C**H**(CH_2Ph)C=O), 5.25-5.12 (m, 4H, COO-C**H**(CH_3)C=O and Ph-C**H2**-O), 4.35 (m, 1H, SiO-C**H**(CH_2Ph)C=O), 3.35-2.75 (m, 4H, Ph-C**H2**-CH), 1.57-1.34 (m, 6H, C**H3**-CH), 0.77 (m, 9H, (C**H3**)$_3$C-Si), -0.20 (m, 6H, $(CH_3)_3$C-Si(C**H3**)$_2$-O) ppm; MS (ESI/Q-TOF): m/z calcd for $C_{37}H_{46}O_9Si+Na^+$ [**M**+Na]$^+$: 685; found 686.

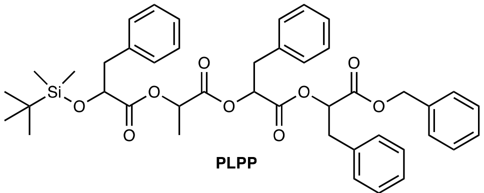

PLPP

PLPP. Colorless oil (6.52 g, 92%); ^{1}H NMR (500MHz, $CDCl_3$): δ 7.37-6.90 (m, 20H, Ph-**H**), 5.42-5.02 (m, 5H, COO-C**H**(CH_2Ph)C=O, COO-C**H**(CH_3)C=O and Ph-C**H2**-O), 4.33 (m, 1H, SiO-C**H**(CH_2Ph) C=O), 3.25-2.70 (m, 6H, Ph-C**H2**-CH), 1.46-1.28 (m, 3H, C**H3**-CH), 0.74 (dd, *J* = 6.1, 3.4 Hz, 9H, (C**H3**)$_3$C-Si), -0.25 (m, 6H, $(CH_3)_3$C-Si(C**H3**)$_2$-O) ppm; MS (ESI/Q-TOF): m/z calcd for $C_{43}H_{50}O_9Si+Na^+$ [**M**+Na]$^+$: 761; found 762.

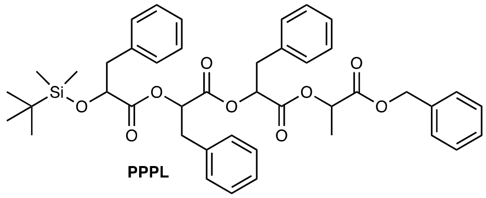

PPPL

PPPL. Colorless oil (2.34 g, 95%); ^{1}H NMR (500MHz, $CDCl_3$): δ 7.50-6.95 (m, 20H, Ph-**H**), 5.45-5.12 (m, 5H, COO-C**H**(CH_2Ph)C=O, COO-C**H**(CH_3)C=O and Ph-C**H2**-O), 4.32 (m, 1H, SiO-C**H**(CH_2Ph) C=O), 3.40-2.60 (m, 6H, Ph-C**H2**-CH), 1.57-1.44 (m, 3H, C**H3**-CH), 0.72 (m, 9H, (C**H3**)$_3$C-Si), -0.30 (m, 6H, $(CH_3)_3$C-Si(C**H3**)$_2$-O) ppm; MS (ESI/Q-TOF): m/z calcd for $C_{43}H_{50}O_9Si+Na^+$ [**M**+Na]$^+$: 761; found 762.

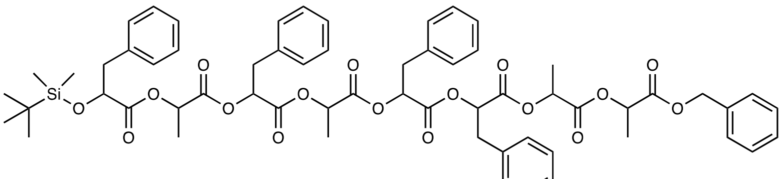

PLPLPPLL

PLPLPPLL (S). Viscous oil (2.1 g, 87%); ^{1}H NMR (500MHz, $CDCl_3$): δ 7.40-6.80 (m, 25H, Ph-**H**), 5.40-5.02 (m, 9H, COO-C**H**(CH_2Ph)C=O, COO-C**H**(CH_3)C=O and Ph-C**H2**-O), 4.32 (m, 1H, SiO-C**H**(CH_2Ph)C=O), 3.40-2.67 (m, 8H, Ph-C**H2**-CH), 1.57-1.17 (m, 12H, C**H3**-CH), 0.75 (m, 9H, (C**H3**)$_3$C-Si), -0.24 (m, 6H, $(CH_3)_3$C-Si(C**H3**)$_2$-O) ppm; M_n and *Đ* (GPC): 1210 Da and 1.03; MS (MALDI-TOF): m/z calcd for $C_{61}H_{70}O_{17}Si+Na^+$ [**M**+Na]$^+$: 1125; found 1126.

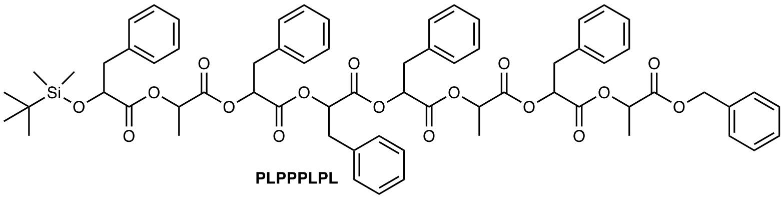

PLPPPLPL

PLPPPLPL (E). Viscous oil (8.37 g,90%); ^{1}H NMR (500MHz, $CDCl_3$): δ 7.40-6.65 (m, 30H, Ph-**H**), 5.43-5.02 (m, 9H, COO-C**H**(CH_2Ph)C=O, COO-C**H**(CH_3)C=O and Ph-C**H2**-O), 4.32 (m, 1H, SiO-

C**H**(CH$_2$Ph)C=O), 3.40-2.67 (m, 10H, Ph-C**H**$_2$-CH), 1.57-1.20 (m, 9H, C**H**$_3$-CH), 0.75 (m, 9H, (C**H**$_3$)$_3$C-Si), -0.24 (m, 6H, (CH$_3$)$_3$C-Si(C**H**$_3$)$_2$-O) ppm; M_n and *Đ* (GPC): 1320 Da and 1.03; MS (MALDI-TOF): m/z calcd for $C_{67}H_{74}O_{17}Si+Na^+$ [**M**+Na]$^+$: 1201; found 1202.

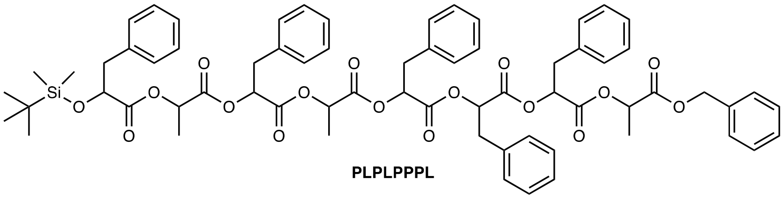

PLPLPPPL

PLPLPPPL (Q). Viscous oil (2.23 g, 92%); ^{1}H NMR (500MHz, CDCl$_3$): δ 7.40-6.65 (m, 30H, Ph-**H**), 5.43-5.02 (m, 9H, COO-C**H**(CH$_2$Ph)C=O, COO-C**H**(CH$_3$)C=O and Ph-C**H**$_2$-O), 4.32 (m, 1H, SiO-C**H**(CH$_2$Ph)C=O), 3.40-2.67 (m, 10H, Ph-C**H**$_2$-CH), 1.57-1.18 (m, 9H, C**H**$_3$-CH), 0.75 (m, 9H, (C**H**$_3$)$_3$C-Si), -0.24 (m, 6H, (CH$_3$)$_3$C-Si(C**H**$_3$)$_2$-O) ppm; M_n and *Đ* (GPC): 1280 Da and 1.03; MS (MALDI-TOF): m/z calcd for $C_{67}H_{74}O_{17}Si+Na^+$ [**M**+Na]$^+$: 1201; found 1202.

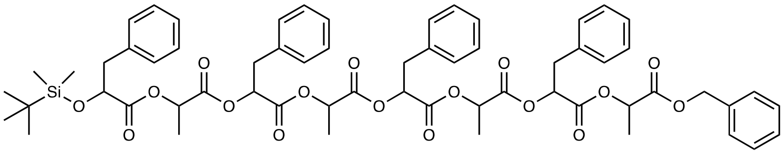

PLPLPLPL

PLPLPLPL (U). Viscous oil (2.35 g, 92%); ^{1}H NMR (500MHz, CDCl$_3$): δ 7.40-7.15 (m, 25H, Ph-**H**), 5.40-5.02 (m, 9H, COO-C**H**(CH$_2$Ph)C=O, COO-C**H**(CH$_3$)C=O and Ph-C**H**$_2$-O), 4.32 (m, 1H, SiO-C**H**(CH$_2$Ph)C=O), 3.40-2.67 (m, 8H, Ph-C**H**$_2$-CH), 1.57-1.20 (m, 12H, C**H**$_3$-CH), 0.75 (m, 9H, (C**H**$_3$)$_3$C-Si), -0.24 (m, 6H, (CH$_3$)$_3$C-Si(C**H**$_3$)$_2$-O) ppm; M_n and *Đ* (GPC): 1240 Da and 1.03; MS (MALDI-TOF): m/z calcd for $C_{61}H_{70}O_{17}Si+Na^+$ [**M**+Na]$^+$: 1125; found 1126.

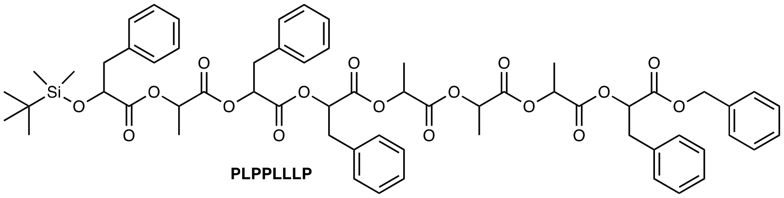

PLPPLLLP

PLPPLLLP (N). Viscous oil (1.75 g, 89%); ^{1}H NMR (500MHz, CDCl$_3$): δ 7.40-6.80 (m, 25H, Ph-**H**), 5.40-5.02 (m, 9H, COO-C**H**(CH$_2$Ph)C=O, COO-C**H**(CH$_3$)C=O and Ph-C**H**$_2$-O), 4.32 (m, 1H, SiO-C**H**(CH$_2$Ph)C=O), 3.40-2.67 (m, 8H, Ph-C**H**$_2$-CH), 1.57-1.20 (m, 12H, C**H**$_3$-CH), 0.75 (m, 9H, (C**H**$_3$)$_3$C-Si), -0.24 (m, 6H, (CH$_3$)$_3$C-Si(C**H**$_3$)$_2$-O) ppm; M_n and *Đ* (GPC): 1210 Da and 1.03; MS (MALDI-TOF): m/z calcd for $C_{61}H_{70}O_{17}Si+Na^+$ [**M**+Na]$^+$: 1125; found 1126.

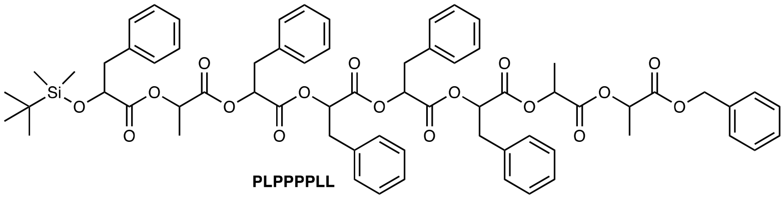

PLPPPPLL

PLPPPPLL (C). Viscous oil (1.80 g, 87%); ^{1}H NMR (500MHz, CDCl$_3$): δ 7.40-6.65 (m, 30H, Ph-**H**), 5.43-5.02 (m, 9H, COO-C**H**(CH$_2$Ph)C=O, COO-C**H**(CH$_3$)C=O and Ph-C**H**$_2$-O), 4.32 (m, 1H, SiO-C**H**(CH$_2$Ph)C=O), 3.40-2.67 (m, 10H, Ph-C**H**$_2$-CH), 1.57-1.18 (m, 9H, C**H**$_3$-CH), 0.75 (m, 9H, (C**H**$_3$)$_3$C-Si), -0.24 (m, 6H, (CH$_3$)$_3$C-Si(C**H**$_3$)$_2$-O) ppm; M_n and *Đ* (GPC): 1260 Da and 1.03; MS (MALDI-TOF): m/z calcd for $C_{67}H_{74}O_{17}Si+Na^+$ [**M**+Na]$^+$: 1201; found 1202.

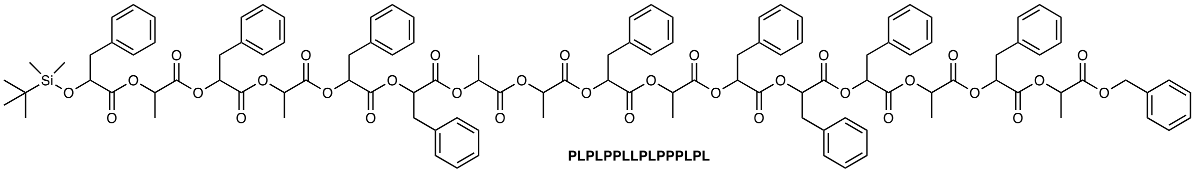

PLPLPPLLPLPPPLPL (SE). A white solid (2.68 g, 83%); ^{1}H NMR (500MHz, $CDCl_3$): δ 7.40-6.65 (m, 50H, Ph-**H**), 5.43-5.02 (m, 17H, COO-C**H**(CH_2Ph)C=O, COO-C**H**(CH_3)C=O and Ph-C$\mathbf{H_2}$-O), 4.32 (m, 1H, SiO-C**H**(CH_2Ph)C=O), 3.40-2.67 (m, 18H, Ph-C$\mathbf{H_2}$-CH), 1.54-1.14 (m, 21H, C$\mathbf{H_3}$-CH), 0.75 (m, 9H, (C$\mathbf{H_3}$)$_3$C-Si), -0.24 (m, 6H, $(CH_3)_3$C-Si(C$\mathbf{H_3}$)$_2$-O) ppm; M_n and $Đ$ (GPC): 2430 Da and 1.01; MS (MALDI-TOF): m/z calcd for $C_{115}H_{122}O_{33}Si+Na^+$ [**M**+Na]$^+$: 2083; found 2083.

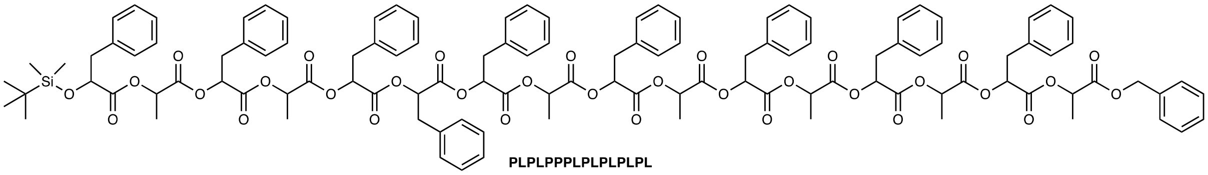

PLPLPPPLPLPLPLPL (QU). A white solid (2.83 g, 83%); ^{1}H NMR (500MHz, $CDCl_3$): δ 7.40-6.65 (m, 50H, Ph-**H**), 5.43-5.02 (m, 17H, COO-C**H**(CH_2Ph)C=O, COO-C**H**(CH_3)C=O and Ph-C$\mathbf{H_2}$-O), 4.32 (m, 1H, SiO-C**H**(CH_2Ph)C=O), 3.40-2.67 (m, 18H, Ph-C$\mathbf{H_2}$-CH), 1.54-1.14 (m, 21H, C$\mathbf{H_3}$-CH), 0.75 (m, 9H, (C$\mathbf{H_3}$)$_3$C-Si), -0.24 (m, 6H, $(CH_3)_3$C-Si(C$\mathbf{H_3}$)$_2$-O) ppm; M_n and $Đ$ (GPC): 2400 Da and 1.01; MS (MALDI-TOF): m/z calcd for $C_{115}H_{122}O_{33}Si+Na^+$ [**M**+Na]$^+$: 2083; found 2083.

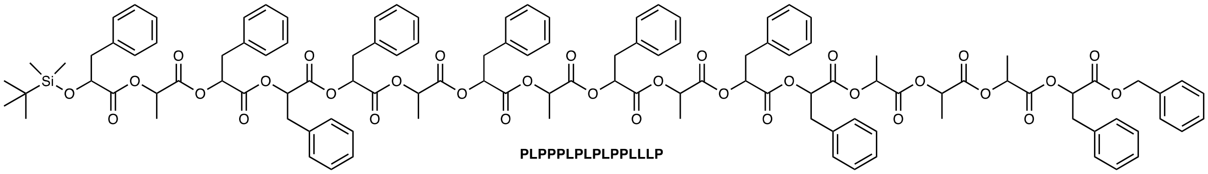

PLPPPLPLPLPPLLLP (EN). A white solid (2.02 g, 74%); ^{1}H NMR (500MHz, $CDCl_3$): δ 7.40-6.65 (m, 50H, Ph-**H**), 5.43-5.02 (m, 17H, COO-C**H**(CH_2Ph)C=O, COO-C**H**(CH_3)C=O and Ph-C$\mathbf{H_2}$-O), 4.32 (m, 1H, SiO-C**H**(CH_2Ph)C=O), 3.40-2.67 (m, 18H, Ph-C$\mathbf{H_2}$-CH), 1.54-1.14 (m, 21H, C$\mathbf{H_3}$-CH), 0.75 (m, 9H, (C$\mathbf{H_3}$)$_3$C-Si), -0.24 (m, 6H, $(CH_3)_3$C-Si(C$\mathbf{H_3}$)$_2$-O) ppm; M_n and $Đ$ (GPC): 2390 Da and 1.01; MS (MALDI-TOF): m/z calcd for $C_{115}H_{122}O_{33}Si+Na^+$ [**M**+Na]$^+$: 2083; found 2083.

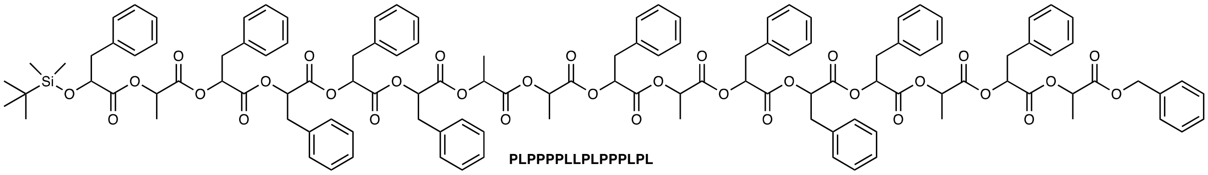

PLPPPPLLPLPPPLPL (CE). A white solid (1.47 g, 81%); ^{1}H NMR (500MHz, $CDCl_3$): δ 7.40-6.65 (m, 55H, Ph-**H**), 5.43-5.02 (m, 17H, COO-C**H**(CH_2Ph)C=O, COO-C**H**(CH_3)C=O and Ph-C$\mathbf{H_2}$-O), 4.32 (m, 1H, SiO-C**H**(CH_2Ph)C=O), 3.40-2.71 (m, 20H, Ph-C$\mathbf{H_2}$-CH), 1.54-1.14 (m, 18H, C$\mathbf{H_3}$-CH), 0.75 (m, 9H, (C$\mathbf{H_3}$)$_3$C-Si), -0.24 (m, 6H, $(CH_3)_3$C-Si(C$\mathbf{H_3}$)$_2$-O) ppm; M_n and $Đ$ (GPC): 2440 Da and 1.01; MS (MALDI-TOF): m/z calcd for $C_{121}H_{126}O_{33}Si+Na^+$ [**M**+Na]$^+$: 2159; found 2159.

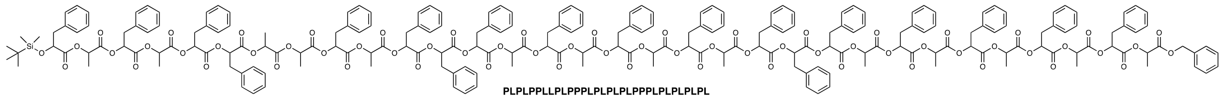

PLPLPPLLPLPPPLPLPLPLPPPLPLPLPLPL (SEQU). A white solid (2.95 g, 71%); ^{1}H NMR (500MHz, $CDCl_3$): δ 7.40-6.65 (m, 95H, Ph-**H**), 5.43-5.02 (m, 33H, COO-C**H**(CH_2Ph)C=O, COO-C**H**(CH_3)C=O and Ph-C$\mathbf{H_2}$-O), 4.32 (m, 1H, SiO-C**H**(CH_2Ph)C=O), 3.40-2.67 (m, 36H, Ph-C$\mathbf{H_2}$-CH),

1.54-1.14 (m, 42H, C**H**$_3$-CH), 0.75 (m, 9H, (C**H**$_3$)$_3$C-Si), -0.24 (m, 6H, (CH$_3$)$_3$C-Si(C**H**$_3$)$_2$-O) ppm; M_n and *Đ* (GPC): 4120 Da and 1.02; MS (MALDI-TOF): m/z calcd for $C_{217}H_{222}O_{65}Si+Na^+$ [**M**+Na]$^+$: 3920; found 3920.

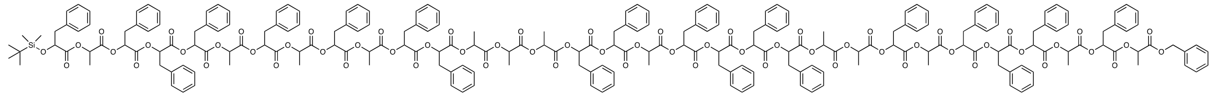

PLPPPLPLPLPPLLLPPLPPPPLLPLPPPLPL

PLPPPLPLPLPPLLLPPLPPPPLLPLPPPLPL (ENCE). A white solid (2.06 g, 88%); ^{1}H NMR (500MHz, CDCl$_3$): δ 7.40-6.65 (m, 100H, Ph-**H**), 5.43-5.02 (m, 33H, COO-C**H**(CH$_2$Ph)C=O, COO-C**H**(CH$_3$)C=O and Ph-C**H$_2$**-O), 4.32 (m, 1H, SiO-C**H**(CH$_2$Ph)C=O), 3.40-2.67 (m, 38H, Ph-C**H$_2$**-CH), 1.54-1.14 (m, 39H, C**H$_3$**-CH), 0.75 (m, 9H, (C**H$_3$**)$_3$C-Si), -0.24 (m, 6H, (CH$_3$)$_3$C-Si(C**H$_3$**)$_2$-O) ppm; M_n and *Đ* (GPC): 4090 Da and 1.02; MS (MALDI-TOF): m/z calcd for $C_{223}H_{226}O_{65}Si+Na^+$ [**M**+Na]$^+$: 3996; found 3996.

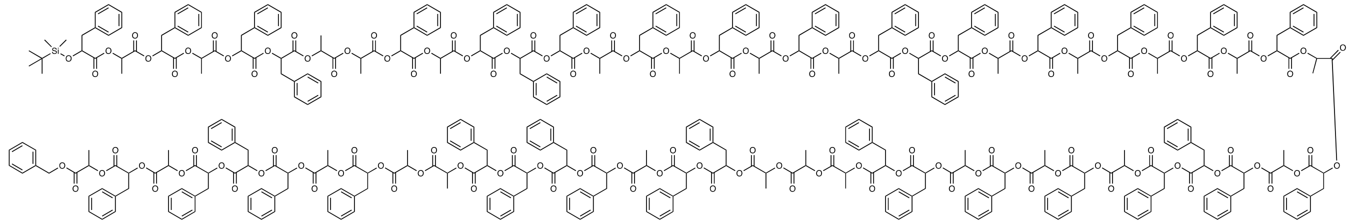

PLPLPPLLPLPPPLPLPLPLPPPLPLPLPLPLPPPLPLPLPPLLLPPLPPPPLLPLPPPLPL (SEQUENCE). A white solid (2.49 g, 71%); ^{1}H NMR (500MHz, CDCl$_3$): δ 7.40-6.65 (m, 190H, Ph-**H**), 5.43-5.02 (m, 65H, COO-C**H**(CH$_2$Ph)C=O, COO-C**H**(CH$_3$)C=O and Ph-C**H$_2$**-O), 4.32 (m, 1H, SiO-C**H**(CH$_2$Ph)C=O), 3.40-2.67 (m, 74H, Ph-C**H$_2$**-CH), 1.54-1.14 (m, 81H, C**H$_3$**-CH), 0.75 (m, 9H, (C**H$_3$**)$_3$C-Si), -0.24 (m, 6H, (CH$_3$)$_3$C-Si(C**H$_3$**)$_2$-O) ppm; M_n and *Đ* (GPC): 8170 Da and 1.03; MS (MALDI-TOF): m/z calcd for $C_{427}H_{426}O_{129}Si+Na^+$ [**M**+Na]$^+$: 7673; found 7674.

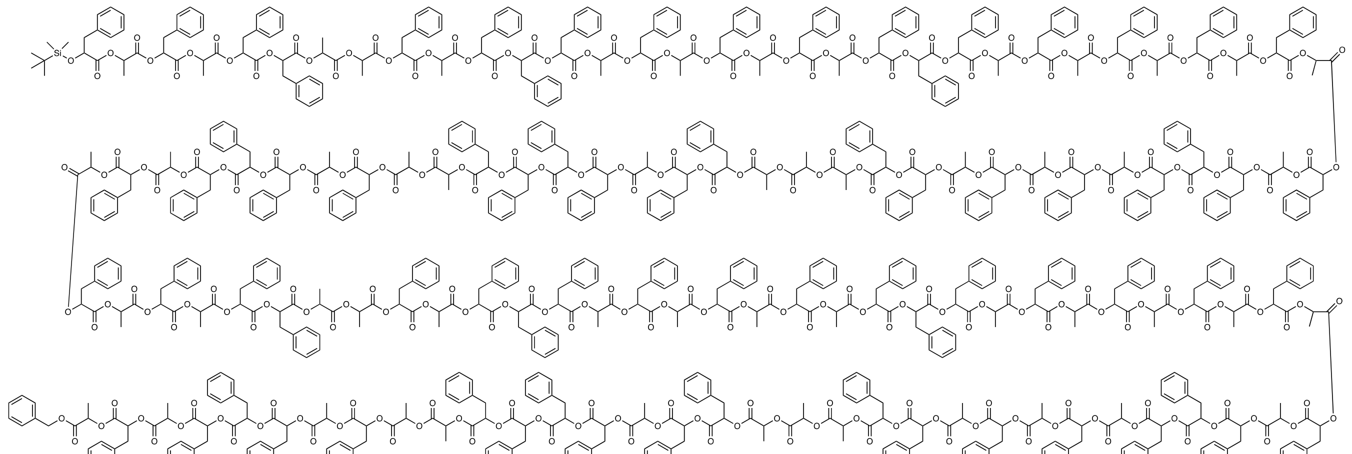

PLPLPPLLPLPPPLPLPLPLPPPLPLPLPLPLPPPLPLPLPPLLLPPLPPPPLLPLPPPLPL PLPLPPLLPLPPPLPLPLPLPPPLPLPLPLPLPPPLPLPLPPLLLPPLPPPPLLPLPPPLPL (SEQUENCESEQUENCE). A white solid (0.63 g, 70%); ^{1}H NMR (500MHz, CDCl$_3$): δ 7.40-6.65 (m, 375H, Ph-**H**), 5.43-5.02 (m, 129H, COO-C**H**(CH$_2$Ph)C=O, COO-C**H**(CH$_3$)C=O and Ph-C**H$_2$**-O), 4.32 (m, 1H, SiO-C**H**(CH$_2$Ph)C=O), 3.40-2.67 (m, 148H, Ph-C**H$_2$**-CH), 1.54-1.14 (m, 162H, C**H$_3$**-CH), 0.75

(m, 9H, $(C\mathbf{H_3})_3C$-Si), -0.24 (m, 6H, $(CH_3)_3C$-Si$(C\mathbf{H_3})_2$-O) ppm; M_n and $Đ$ (GPC): 16230 Da and 1.03; MS (MALDI-TOF): molecular m/z calcd for $C_{841}H_{830}O_{257}Si+Na^+$ $[\mathbf{M}+Na]^+$: 15101; found 15105.

Synthesis of poly(α-hydroxy acid) (PAH)

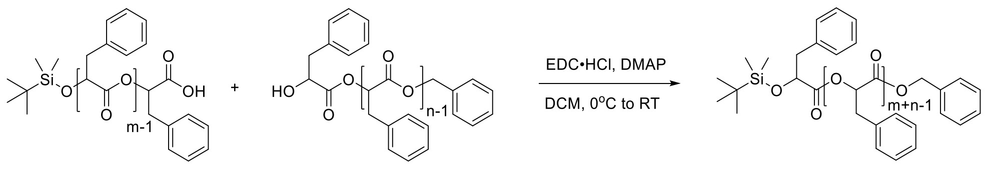

TBDMS-PA$_m$-COOH HO-PA$_n$-Bz **PA(m+n)**

TBDMS-PA$_m$-COOH was prepared by the hydrogenation of PAm according to the described general procedure. HO-PA$_n$-Bz was prepared by removing the TBDMS group from PAn according to the described general procedure. An equimolar mixture of TBDMS-PA$_m$-COOH and HO-PA$_n$-Bz was reacted with EDC•HCl in the presence of DMAP (5 mol%) in dry DCM. The reaction time varied from 5 h to 24 h depending on m and n. The desired product PA(m+n) was isolated by automated column chromatography using n-hexane/EA (19:1 to 4:1 v/v) as an eluent. PPA with 32 repeating units or more was purified by size exclusion chromatography with a series of columns. Chloroform was used as an eluent.

PA4. Colorless oil (10g, 93%); ^{1}H NMR (500MHz, $CDCl_3$): 7.37-7.27 (m, 5H, Ph-**H**), 5.51-5.18 (m, 3H, COO-C**H**(CH_3)C=O), 5.13 (m, 2H, Ph-C$\mathbf{H_2}$-O), 4.27 (ddd, J = 16.1, 10.9, 7.8 Hz, 1H, SiO-C**H**(CH_3)C=O), 3.36-2.49 (m, 6H, C$\mathbf{H_3}$-CH), 0.72 (dt, J = 5.0, 2.0 Hz, 9H, $(C\mathbf{H_3})_3C$-Si), -0.30 (ddd, J = 37.1, 32.5, 7.6 Hz, 6H, $(CH_3)_3C$-Si$(C\mathbf{H_3})_2$-O) ppm; ^{13}C NMR (400MHz, $CDCl_3$): δ 172.4 (SiO-CH(CH_2Ph)**C**=O), 168.7 (COO-CH(CH_2Ph)**C**=O), 137.7 ($\mathbf{C_5}H_5$-CH_2-COO), 135.5 ($\mathbf{C_5}H_5$-CH_2-CH-SiO), 135.1 ($\mathbf{C_5}H_5$-CH_2-CH-COO), 129.3 ($\mathbf{C_5}H_5$-CH_2-CH-SiO, $\mathbf{C_5}H_5$-CH_2-CH-COO and $\mathbf{C_5}H_5$-CH_2-COO), 128.6 ($\mathbf{C_5}H_5$-CH_2-CH-SiO, $\mathbf{C_5}H_5$-CH_2-CH-COO and $\mathbf{C_5}H_5$-CH_2-COO), 127.2 ($\mathbf{C_5}H_5$-CH_2-CH-SiO, $\mathbf{C_5}H_5$-CH_2-CH-COO and $\mathbf{C_5}H_5$-CH_2-COO), 74.0-73.1 (Ph-CH_2-**C**H-SiO), 72.9-72.4 (Ph-CH_2-**C**H-COO), 67.3 (C_5H_5-$\mathbf{C}H_2$-COO), 41.2 (Ph-$\mathbf{C}H_2$-CH-SiO), 37.1 (Ph-$\mathbf{C}H_2$-CH-COO), 25.6 ($(\mathbf{C}H_3)_3C$-Si$(CH_3)_2$-O), 18.2 ($(CH_3)_3\mathbf{C}$-Si$(CH_3)_2$-O), -5.3 ($(CH_3)_3C$-Si$(\mathbf{C}H_3)_2$-O), -5.9 ($(CH_3)_3C$-Si$(\mathbf{C}H_3)_2$-O) ppm.

PA8. Viscous oil (7.25 g, 84%); ^{1}H NMR (500MHz, $CDCl_3$): 7.37-6.75 (m, 45H, Ph-**H**), 5.52-5.21 (m, 7H, COO-C**H**(CH_2Ph)C=O), 5.12 (m, 2H, Ph-C$\mathbf{H_2}$-O), 4.27 (dd, J = 45.4, 9.2 Hz, 1H, SiO-C**H**(CH_2Ph)C=O), 3.30-2.55 (m, 16H, Ph-C$\mathbf{H_2}$-CH), 0.71 (m, 9H, $(C\mathbf{H_3})_3C$-Si), -0.30 (m, 6H, $(CH_3)_3C$-Si$(C\mathbf{H_3})_2$-O) ppm; MS (MALDI-TOF): m/z (isotopic maximum value) calcd for $C_{85}H_{86}O_{17}Si+Na^+$ $[\mathbf{M}+Na]^+$: 1430; found 1430.

PA16. A white solid (5.41 g, 81%); ^{1}H NMR (400MHz, $CDCl_3$): 7.43-6.73 (m, 85H, Ph-z**H**), 5.50-5.221 (m, 15H, COO-C**H**(CH_2Ph)C=O), 5.13 (m, 2H, Ph-C$\mathbf{H_2}$-O), 4.30 (dd, J = 31.3, 23.6 Hz, 1H, SiO-C**H**(CH_2Ph)C=O), 3.32-2.53 (m, 32H, Ph-C$\mathbf{H_2}$-CH), 0.73 (m, 9H, $(C\mathbf{H_3})_3C$-Si), -0.30 (m, 6H, $(CH_3)_3C$-Si$(C\mathbf{H_3})_2$-O) ppm; M_n and $Đ$ (GPC): 2590 Da and 1.01; MS (MALDI-TOF): m/z calcd for $C_{157}H_{150}O_{33}Si+Na^+$ $[\mathbf{M}+Na]^+$: 2615; found 2615.

PA32. A white solid (4.30 g, 83%); ^{1}H NMR (500MHz, $CDCl_3$): 7.40-6.80 (m, 165H, Ph-**H**), 5.47-5.22 (m, 31H, COO-C**H**(CH_2Ph)C=O), 5.16 (m, 2H, Ph-C**H₂**-O), 4.30 (m, 1H, SiO-C**H**(CH_2Ph)C=O), 3.35-2.60 (m, 64H, Ph-C**H₂**-CH), 0.76 (m, 9H, (C**H₃**)₃C-Si), -0.27 (m, 6H, (CH_3)$_3$C-Si(C**H₃**)₂-O) ppm; M_n and *Đ* (GPC): 4140 Da and 1.02; MS (MALDI-TOF): m/z calcd for $C_{301}H_{278}O_{65}Si+Na^+$ [**M**+Na]$^+$: 4985; found 4986.

PA64. A white solid (3.11 g, 74%); ^{1}H NMR (500MHz, $CDCl_3$): 7.40-6.80 (m, 325H, Ph-**H**), 5.40-5.17 (m, 63H, COO-C**H**(CH_2Ph)C=O), 5.13 (m, 2H, Ph-C**H₂**-O), 4.25 (m, 1H, SiO-C**H**(CH_2Ph)C=O), 3.30-2.74 (m, 128H, Ph-C**H₂**-CH), 0.71 (m, 9H, (C**H₃**)₃C-Si), -0.30 (m, 6H, (CH_3)$_3$C-Si(C**H₃**)₂-O) ppm; M_n and *Đ* (GPC): 8670 Da and 1.02; MS (MALDI-TOF): m/z calcd for $C_{589}H_{534}O_{129}Si+Na^+$ [**M**+Na]$^+$: 9726; found 9728.

PA80. A white solid (0.22g, 80%); ^{1}H NMR (500MHz, $CDCl_3$): 7.40-6.80 (m, 325H, Ph-**H**), 5.40-5.17 (m, 79H, COO-C**H**(CH_2Ph)C=O), 5.13 (m, 2H, Ph-C**H₂**-O), 4.25 (m, 1H, SiO-C**H**(CH_2Ph)C=O), 3.30-2.74 (m, 160H, Ph-C**H₂**-CH), 0.71 (m, 9H, (C**H₃**)₃C-Si), -0.30 (m, 6H, (CH_3)$_3$C-Si(C**H₃**)₂-O) ppm; M_n and *Đ* (GPC): 11860 Da and 1.02; MS (MALDI-TOF): molecular m/z calcd for $C_{733}H_{662}O_{161}Si+Na^+$ [**M**+Na]$^+$: 12098; found 12101.

PA96. A white solid (0.19 g, 77%); ^{1}H NMR (500MHz, $CDCl_3$): 7.40-6.80 (m, 325H, Ph-**H**), 5.40-5.17 (m, 95H, COO-C**H**(CH_2Ph)C=O), 5.13 (m, 2H, Ph-C**H₂**-O), 4.25 (m, 1H, SiO-C**H**(CH_2Ph)C=O), 3.30-2.74 (m, 192H, Ph-C**H₂**-CH), 0.71 (m, 9H, (C**H₃**)₃C-Si), -0.30 (m, 6H, (CH_3)$_3$C-Si(C**H₃**)₂-O) ppm; M_n and *Đ* (GPC): 14380 Da and 1.03; MS (MALDI-TOF): molecular m/z calcd for $C_{877}H_{790}O_{193}Si+Na^+$ [**M**+Na]$^+$: 14469; found 14474.

PA128. A white solid (2.03 g, 66%); ^{1}H NMR (500MHz, $CDCl_3$): 7.40-6.80 (m, 645H, Ph-**H**), 5.40-5.17 (m, 127H, COO-C**H**(CH_2Ph)C=O), 5.13 (m, 2H, Ph-C**H₂**-O), 4.25 (m, 1H, SiO-C**H**(CH_2Ph)C=O), 3.30-2.74 (m, 256H, Ph-C**H₂**-CH), 0.71 (m, 9H, (C**H₃**)₃C-Si), -0.30 (m, 6H, (CH_3)$_3$C-Si(C**H₃**)₂-O) ppm; M_n and *Đ* (GPC): 17310 Da and 1.03; MS (MALDI-TOF): molecular m/z calcd for $C_{1165}H_{1046}O_{257}Si+Na^+$ [**M**+Na]$^+$: 19210; found 19221.

PA256. A white solid (1.07 g, 53%); ^{1}H NMR (500MHz, $CDCl_3$): 7.40-6.80 (m, 325H, Ph-**H**), 5.40-5.17 (m, 63H, COO-C**H**(CH_2Ph)C=O), 5.13 (m, 2H, Ph-C**H₂**-O), 4.25 (m, 1H, SiO-C**H**(CH_2Ph)C=O), 3.30-2.74 (m, 128H, Ph-C**H₂**-CH), 0.71 (m, 9H, (C**H₃**)₃C-Si), -0.30 (m, 6H, (CH_3)$_3$C-Si(C**H₃**)₂-O) ppm; M_n and *Đ* (GPC): 32410 Da and 1.03; MS (MALDI-TOF): molecular m/z calcd for $C_{2317}H_{2070}O_{513}Si+Na^+$ [**M**+Na]$^+$: 38174; found 38191.

Separation of PAHs by SEC

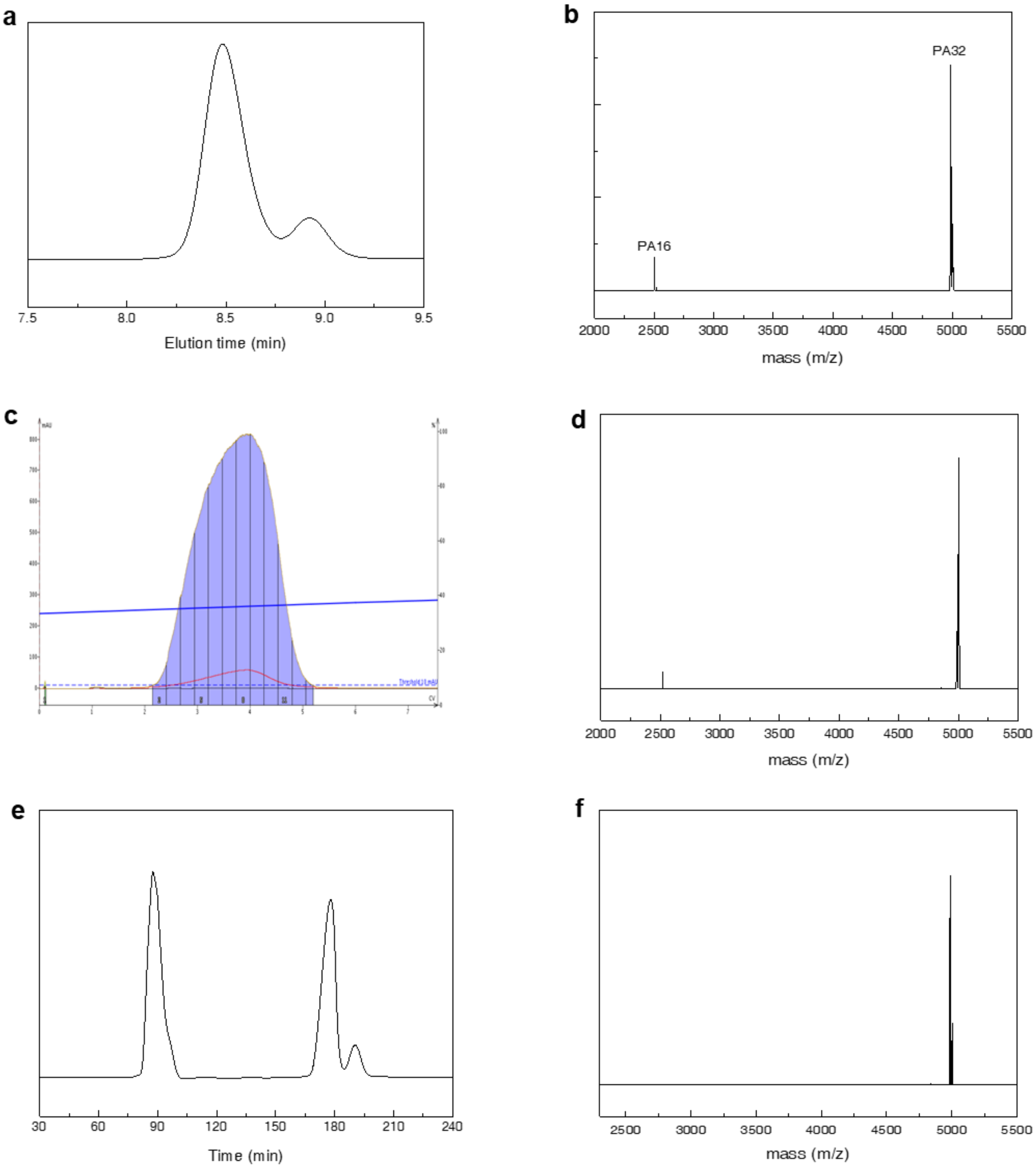

Supplementary Figure 1. a and b, Gel permeation chromatography and MALDI-TOF mass spectra of the crude reaction mixture of **PA32**. **c and d,** UV-detector trace of the automated column chromatography of the crude mixture (c) and MALDI-TOF mass spectrum after purification (d) **e and f,** SEC traces of the crude mixture during the recycling (e) and MALDI-TOF mass spectrum of **PA32** after SEC (f).

GPC measurements of PA*n* and PcL

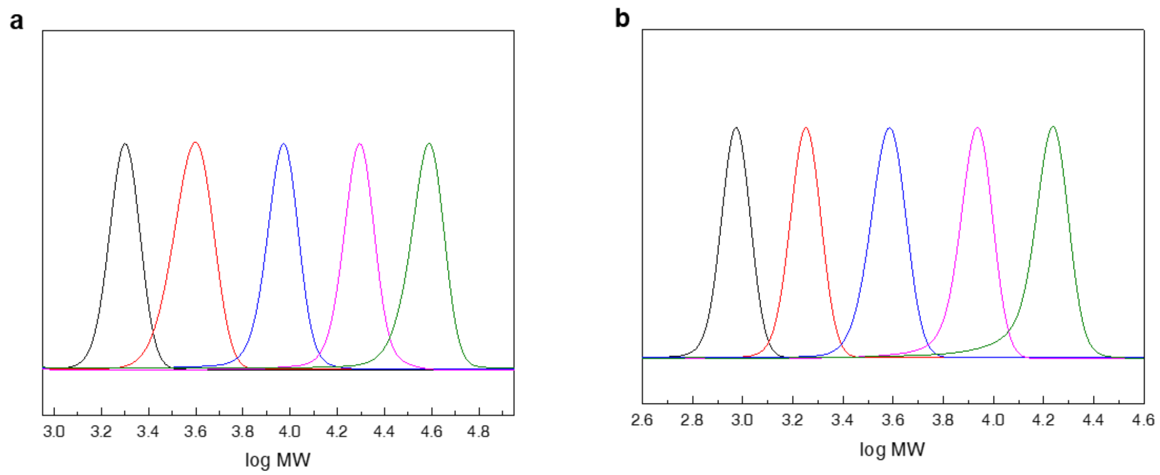

Supplementary Figure 2. GPC peaks of (a) **PAn** (n = 16, 32, 64, 128, 256) and (b) PcL (8 bit, 16 bit, 32 bit, 64 bit, and 128 bit).

NMR spectra of PcLs

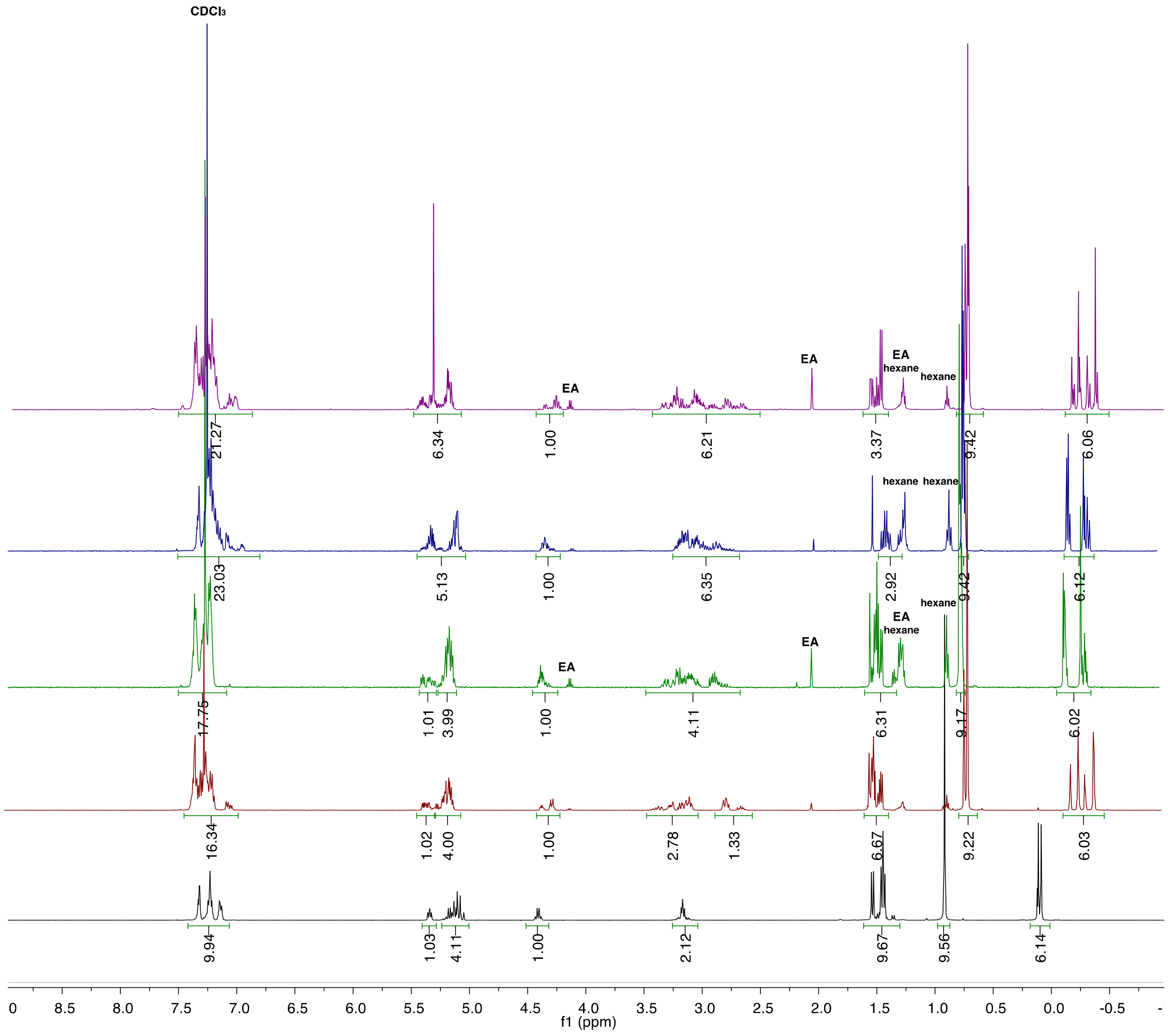

Supplementary Figure 3. ^{1}H NMR spectra of tetrads of PcL. **LLLP** (black), **PPLL** (red), **PLPL** (green), **PLPP** (blue), and **PPPL** (violet).

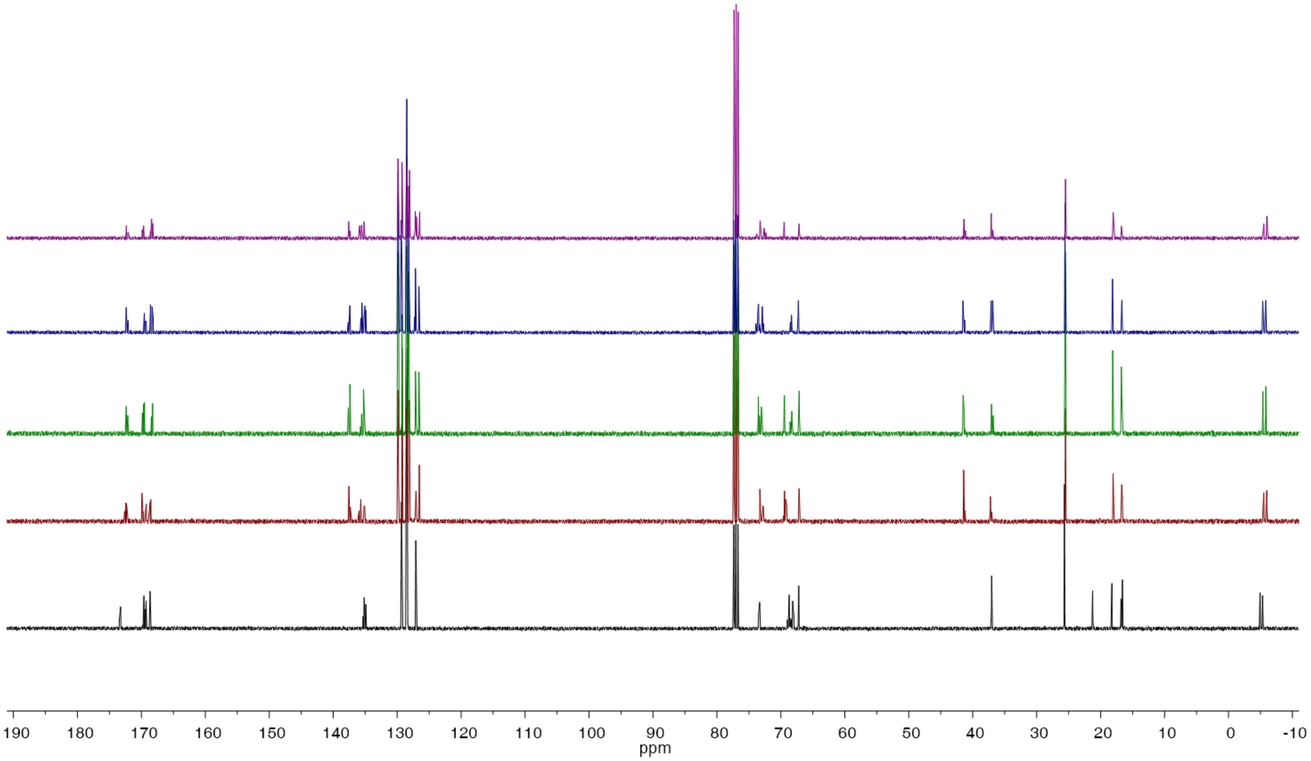

Supplementary Figure 4. ^{13}C NMR spectra of tetrads of PcL. **LLLP** (black), **PPLL** (red), **PLPL** (green), **PLPP** (blue), and **PPPL** (violet).

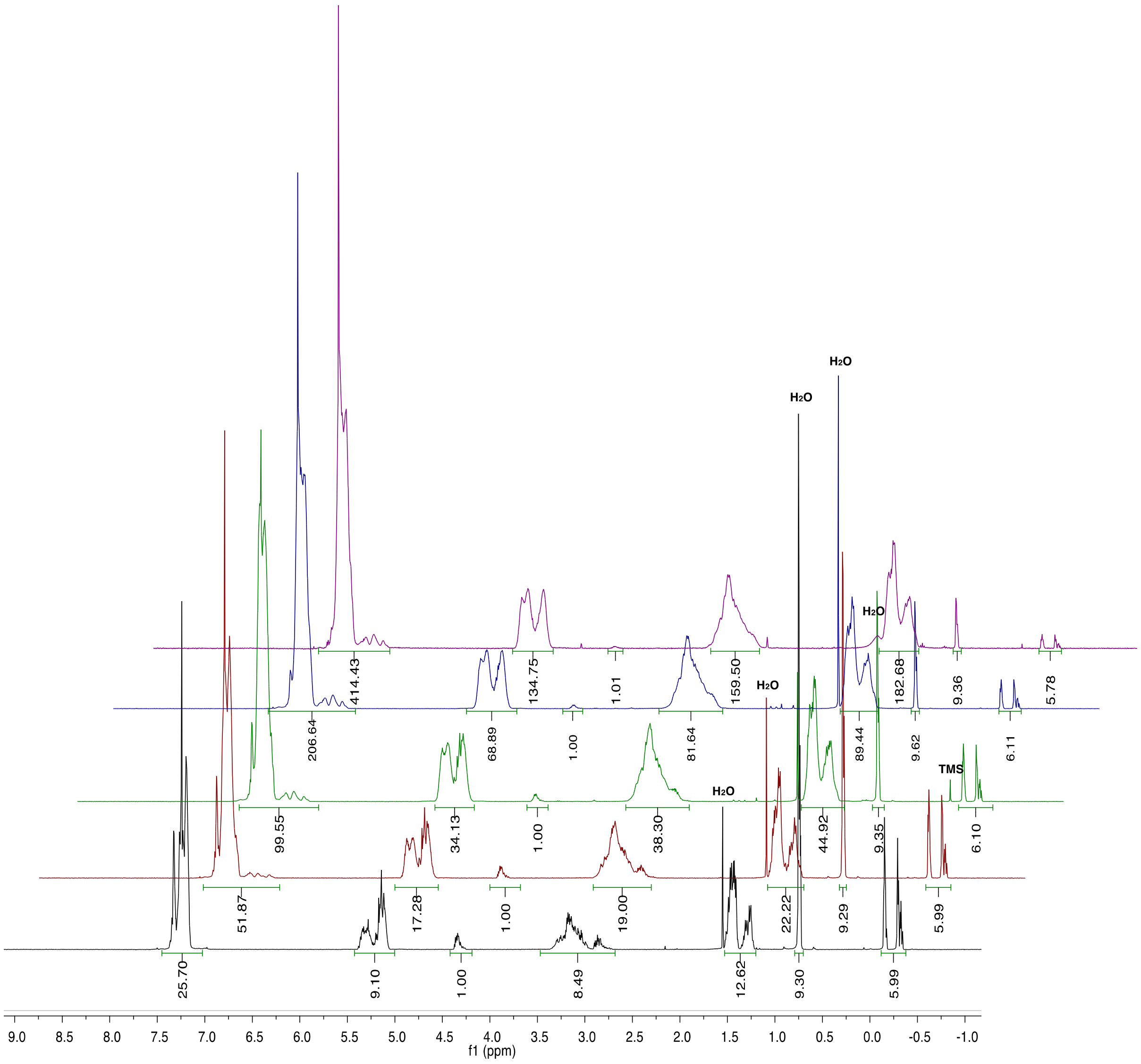

Supplementary Figure 5. ^{1}H NMR spectra of PcLs. **U** (black), **QU** (red), **SEQU** (green), **SEQUENCE** (blue), and **SEQUENCESEQUENCE** (violet).

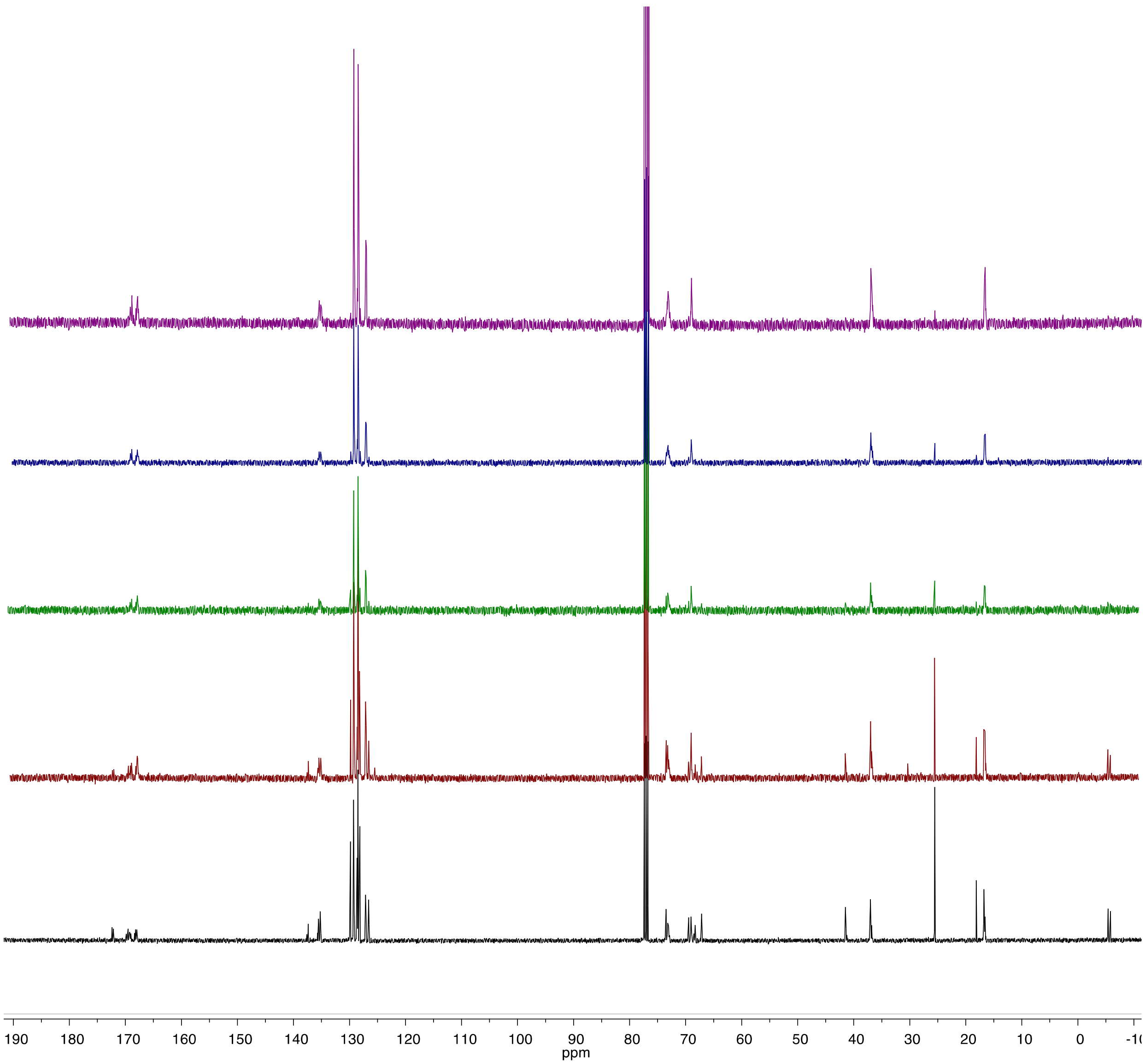

Supplementary Figure 6. ^{13}C NMR spectra of PcLs. **U** (black), **QU** (red), **SEQU** (green), **SEQUENCE** (blue), and **SEQUENCESEQUENCE** (violet).

MALDI-TOF MS/MS Sequencing of PcLs

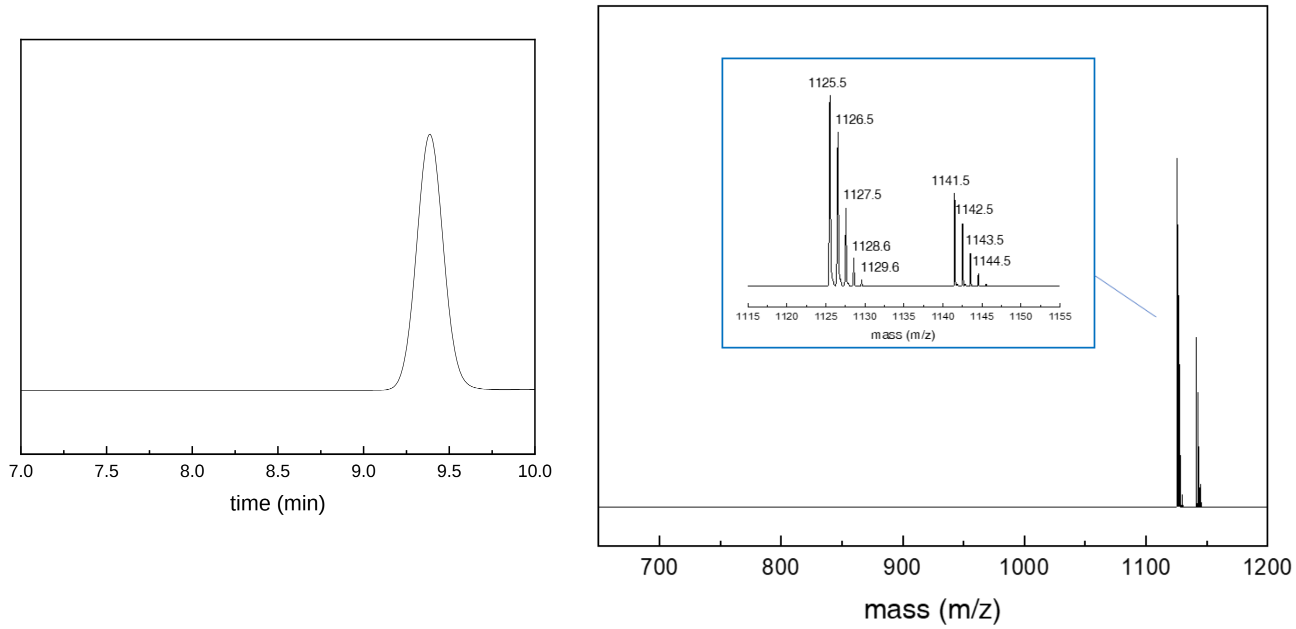

Supplementary Figure 7. GPC and MALDI-TOF MS spectra of PcL (8-bit, S) (theoretical isotopic maximum mass $[M+Na]^+$ = 1125.4 Da).

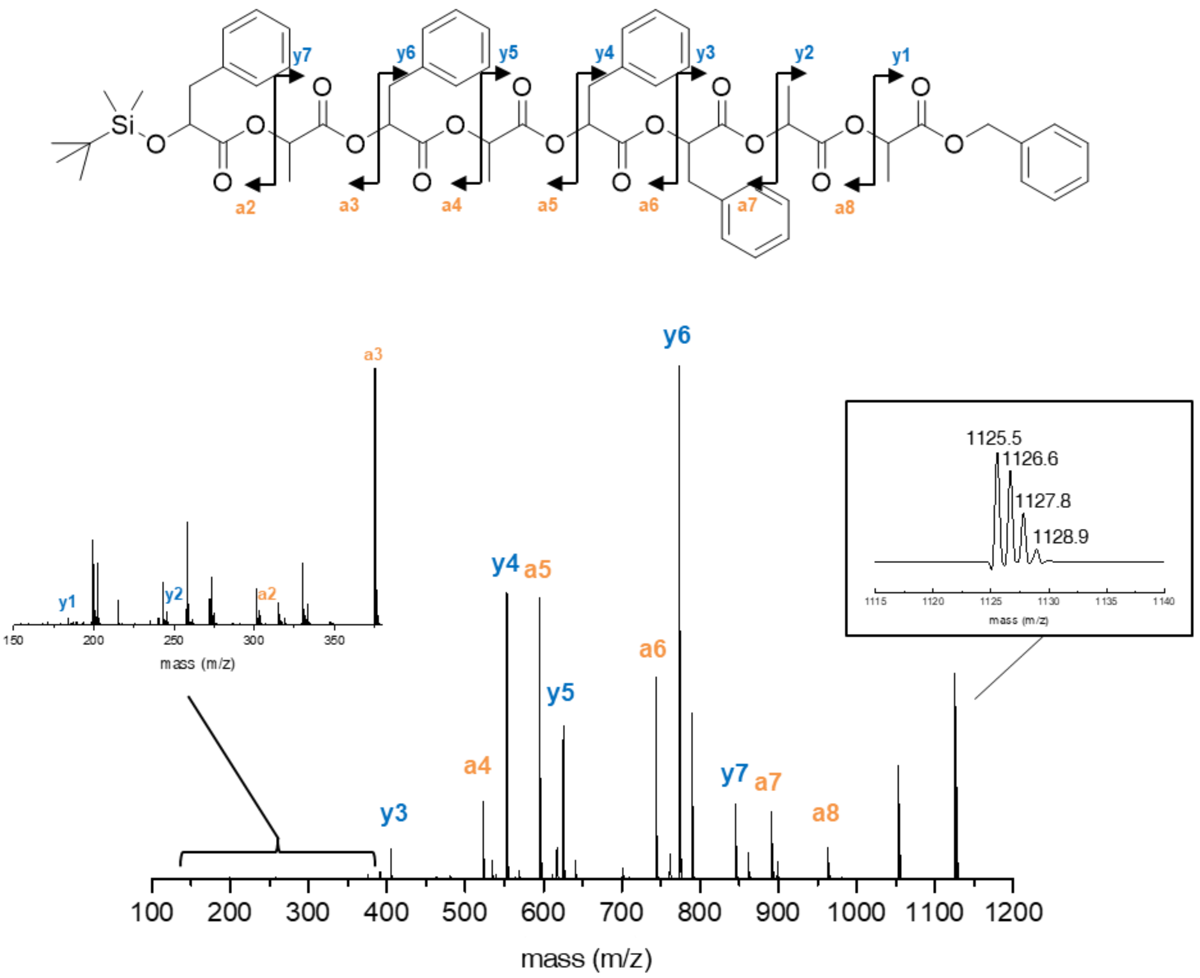

Supplementary Figure 8. MALDI-MS/MS spectrum with peak assignment of PcL (8-bit, S)

Supplementary Table 1. Decoding table of PcL (8-bit, S)

S (PLPLPPLL)									
Si → Bz	Calculated m/z	Found m/z	Difference	Sequence	Bz → Si	Calculated m/z	Found m/z	Difference	Sequence
[M+Na]$^+$	1125.4	1125.5			[M+Na]$^+$	1125.4	1125.5		
y7	845.3	845.5	280.0	Si-P	a8	963.4	963.5	162.0	Bz-L
y6	773.3	773.5	72.0	L	a7	891.3	891.5	72.0	L
y5	625.2	625.5	148.0	P	a6	743.3	743.5	148.0	P
y4	553.2	553.4	72.1	L	a5	595.2	595.5	148.0	P
y3	405.1	405.3	148.1	P	a4	523.2	523.5	72.0	L
y2	257.1	257.3	148.0	P	a3	375.2	375.4	148.1	P
y1	185.1	185.2	72.1	L	a2	303.1	303.4	72.0	L

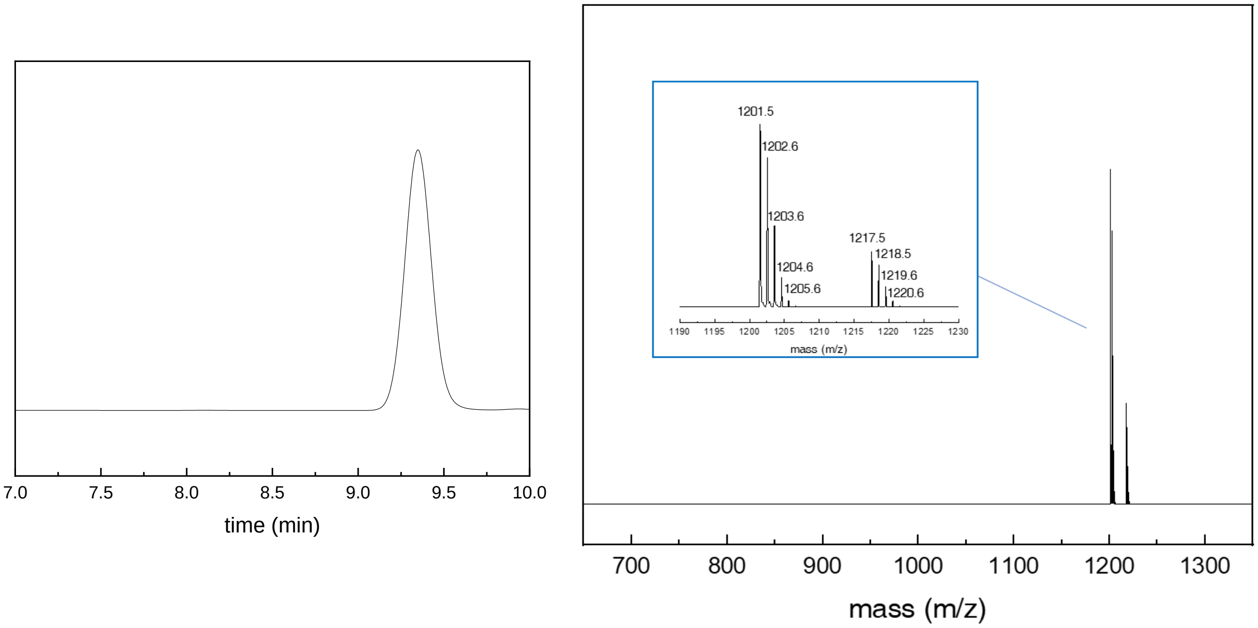

Supplementary Figure 9. GPC and MALDI-TOF MS spectra of PcL (8-bit, E) (theoretical isotopic maximum mass $[M+Na]^+$ = 1201.5 Da).

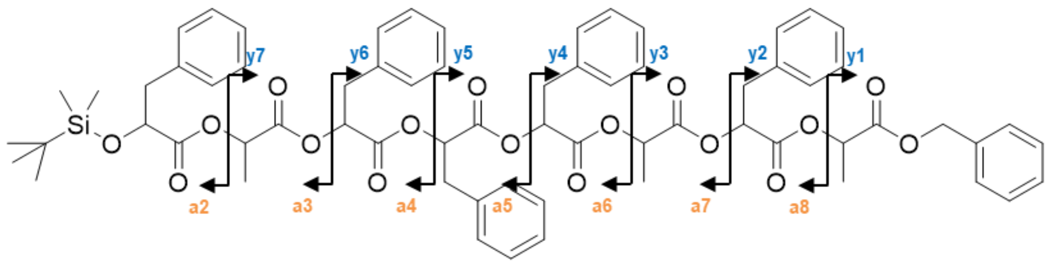

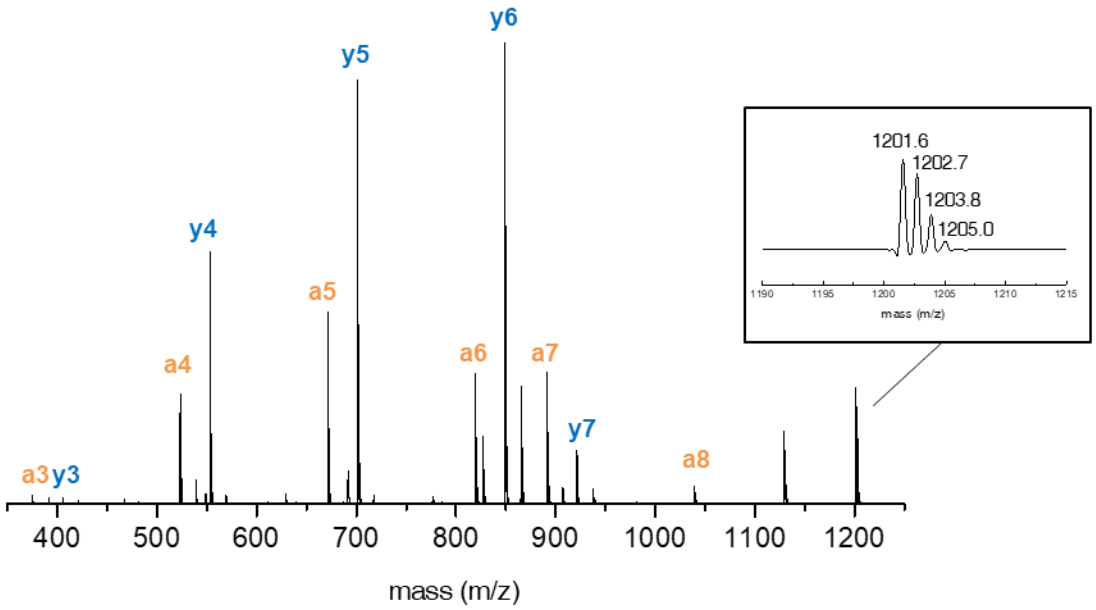

Supplementary Figure 10. MALDI-MS/MS spectrum with peak assignment of PcL (8-bit, E)

Supplementary Table 2. Decoding table of PcL (8-bit, E)

E (PLPPPLPL)									
Si → Bz	Calculated m/z	Found m/z	Difference	Sequence	Bz → Si	Calculated m/z	Found m/z	Difference	Sequence
[M+Na]+	1201.5	1201.5			[M+Na]+	1201.5	1201.5		
y7	921.3	921.4	280.1	Si-P	a8	1039.4	1039.5	162.0	Bz-L
y6	849.3	849.4	72.0	L	a7	891.3	891.4	148.1	P
y5	701.2	701.4	148.0	P	a6	819.3	819.5	71.9	L
y4	553.2	553.4	148.0	P	a5	672.3	671.4	148.1	P
y3	405.1	405.3	148.1	P	a4	523.2	523.4	148.0	P
y2	333.1	333.3	72.0	L	a3	375.2	375.3	148.1	P
y1	185.1	185.2	148.1	P	a2	303.1	303.3	72.0	L

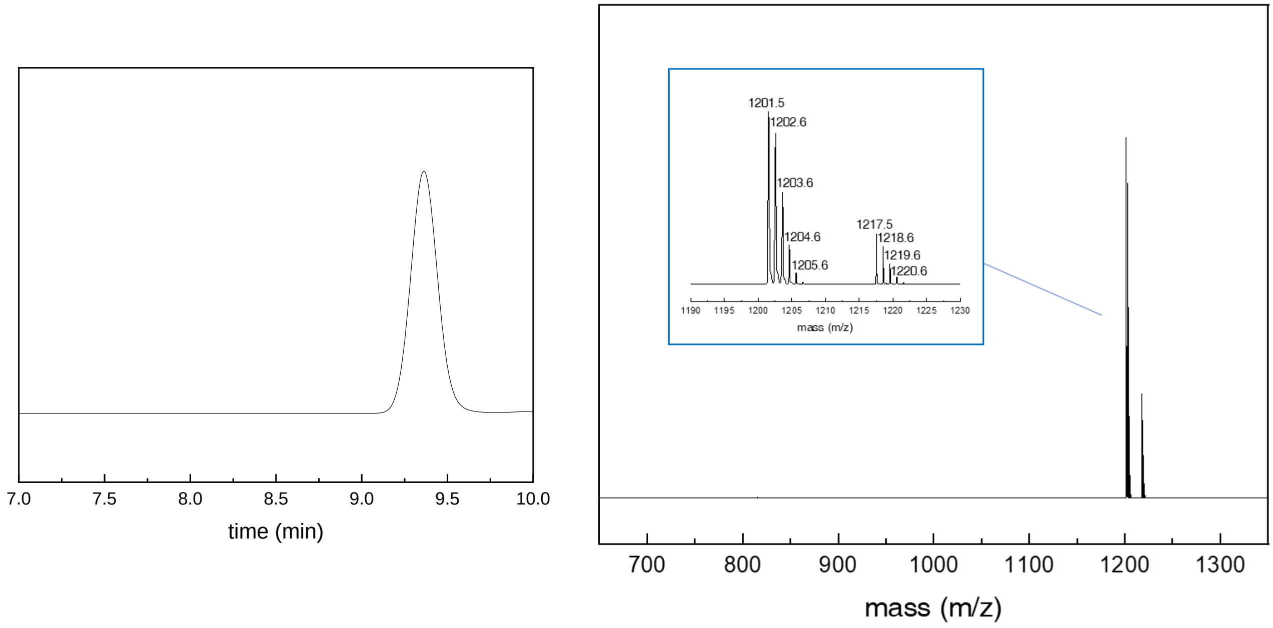

Supplementary Figure 11. GPC and MALDI-TOF MS spectra of PcL (8-bit, Q) (theoretical isotopic maximum mass $[M+Na]^+$ = 1201.5 Da).

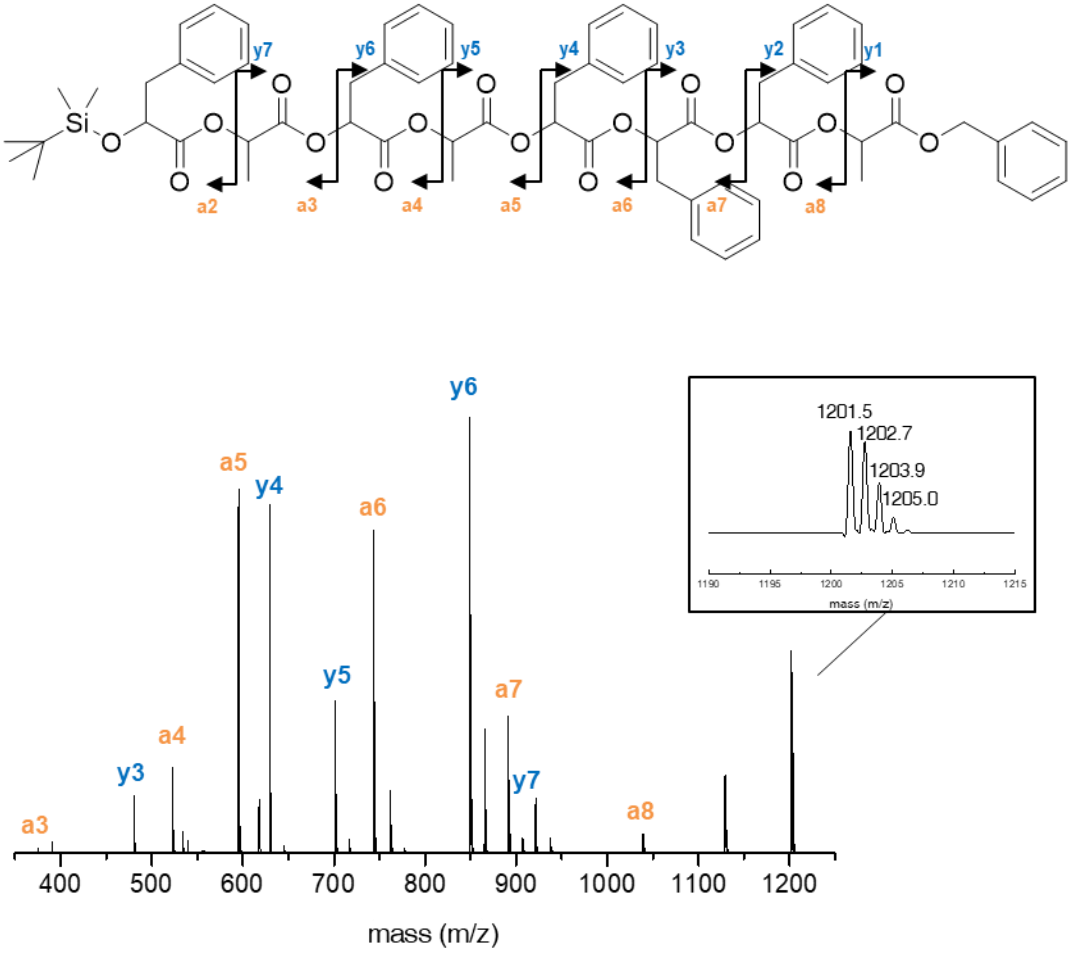

Supplementary Figure 12. MALDI-MS/MS spectrum with peak assignment of PcL (8-bit, Q)

Supplementary Table 3. Decoding table of PcL (8-bit, Q)

Q (PLPLPPPL)									
**Si → Bz**	**Calculated m/z**	**Found m/z**	**Difference**	**Sequence**	**Bz → Si**	**Calculated m/z**	**Found m/z**	**Difference**	**Sequence**
**$[M+Na]^+$**	1201.5	1201.5			**$[M+Na]^+$**	1201.5	1201.5		
**y7**	921.3	921.4	280.1	Si-P	**a8**	1039.4	1039.6	161.9	Bz-L
**y6**	849.3	849.4	72.0	L	**a7**	891.3	891.4	148.2	P
**y5**	701.2	701.4	148.0	P	**a6**	743.3	743.4	148.0	P
**y4**	629.2	629.3	72.1	L	**a5**	595.2	595.4	148.0	P
**y3**	481.2	481.3	148.0	P	**a4**	523.2	523.4	72.0	L
**y2**	333.1	333.2	148.1	P	**a3**	375.2	375.3	148.1	P
**y1**	185.1	185.2	148.0	P	**a2**	303.1	303.3	72.0	L

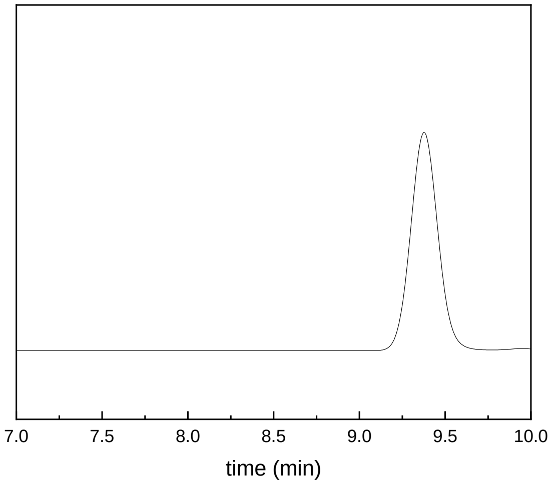

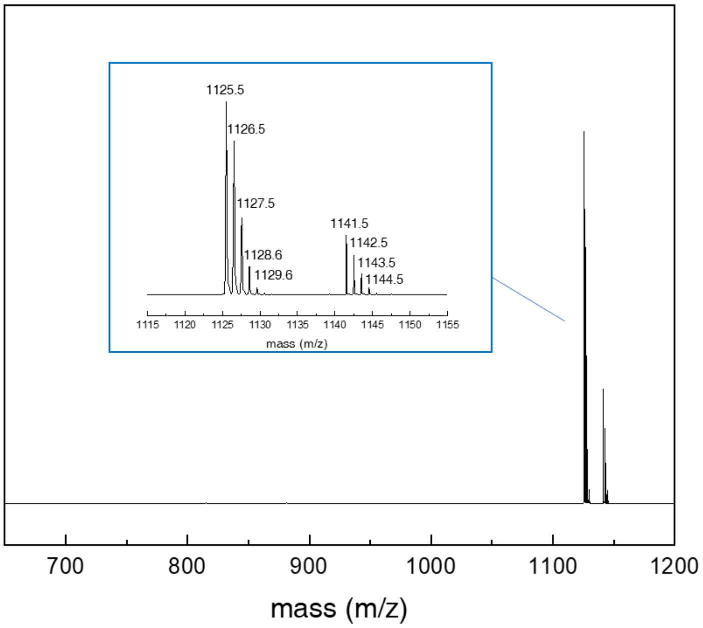

Supplementary Figure 13. GPC and MALDI-TOF MS spectra of PcL (8-bit, U) (theoretical isotopic maximum mass $[M+Na]^+$ = 1125.4 Da).

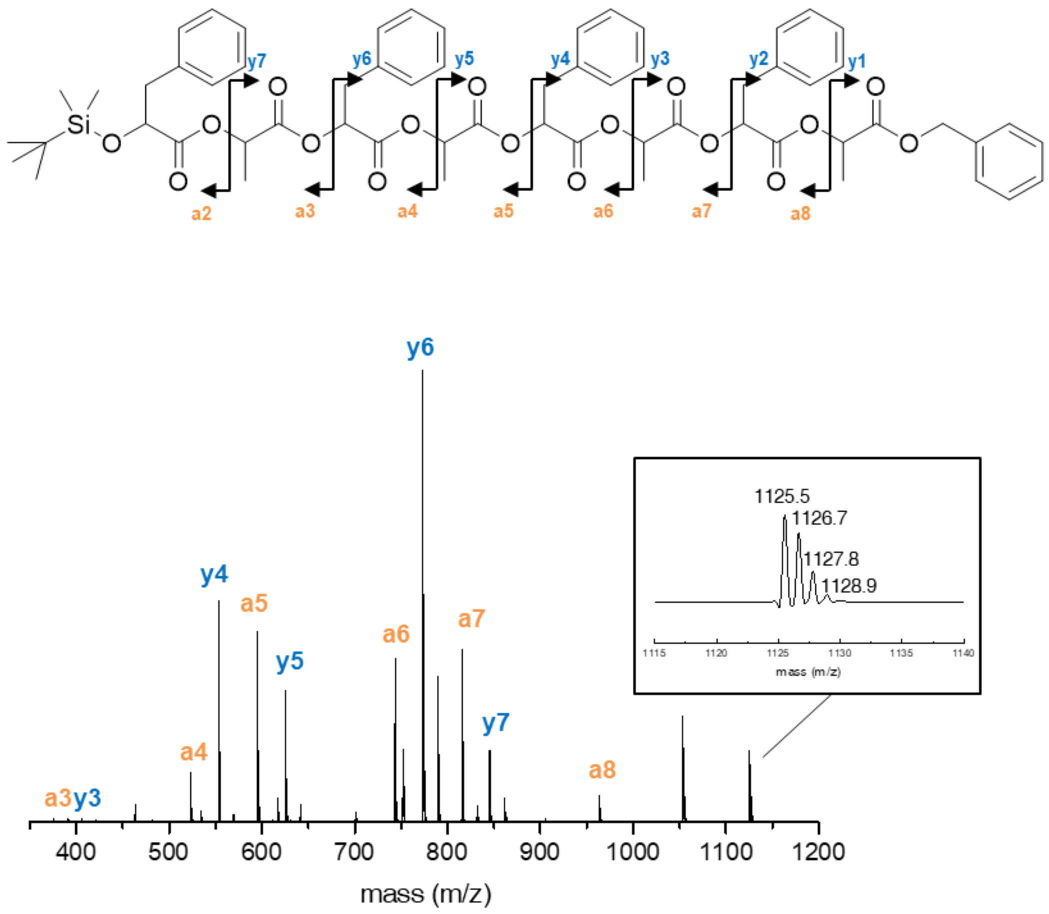

Supplementary Figure 14. MALDI-MS/MS spectrum with peak assignment of PcL (8-bit, U)

Supplementary Table 4. Decoding table of PcL (8-bit, U)

U (PLPLPLPL)

Si → Bz	Calculated m/z	Found m/z	Difference	Sequence	Bz → Si	Calculated m/z	Found m/z	Difference	Sequence
$[M+Na]^+$	1125.4	1125.5			$[M+Na]^+$	1125.4	1125.5		
y7	845.3	845.5	280.0	Si-P	a8	963.4	963.5	162.0	Bz-L
y6	773.3	773.5	72.0	L	a7	815.3	815.4	148.1	P
y5	625.2	625.4	148.1	P	a6	743.3	743.5	71.9	L
y4	553.2	553.4	72.0	L	a5	595.2	595.5	148	P
y3	405.1	405.4	148.0	P	a4	523.2	523.5	72.0	L
y2	333.1	333.4	72.0	L	a3	375.2	375.4	148.1	P
y1	185.1	185.2	148.2	P	a2	303.1	303.3	72.1	L

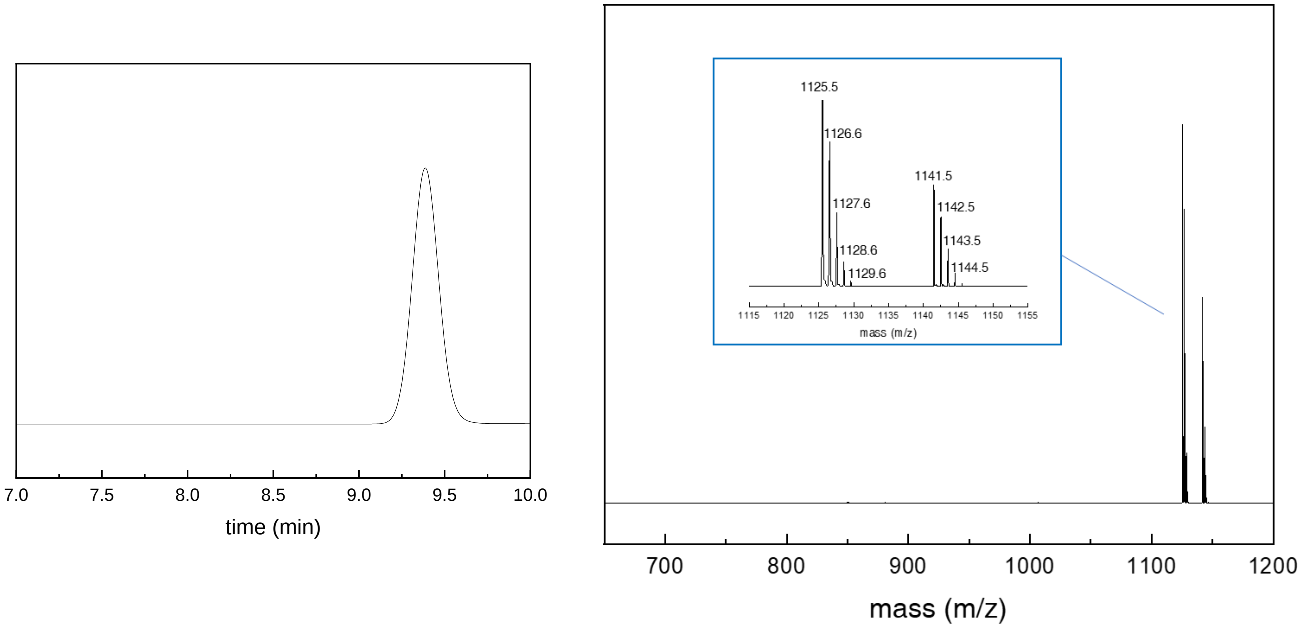

Supplementary Figure 15. GPC and MALDI-TOF MS spectra of PcL (8-bit, N) (theoretical isotopic maximum mass $[M+Na]^+$ = 1125.4 Da).

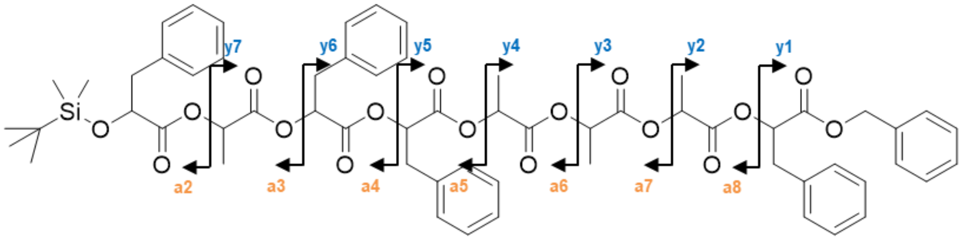

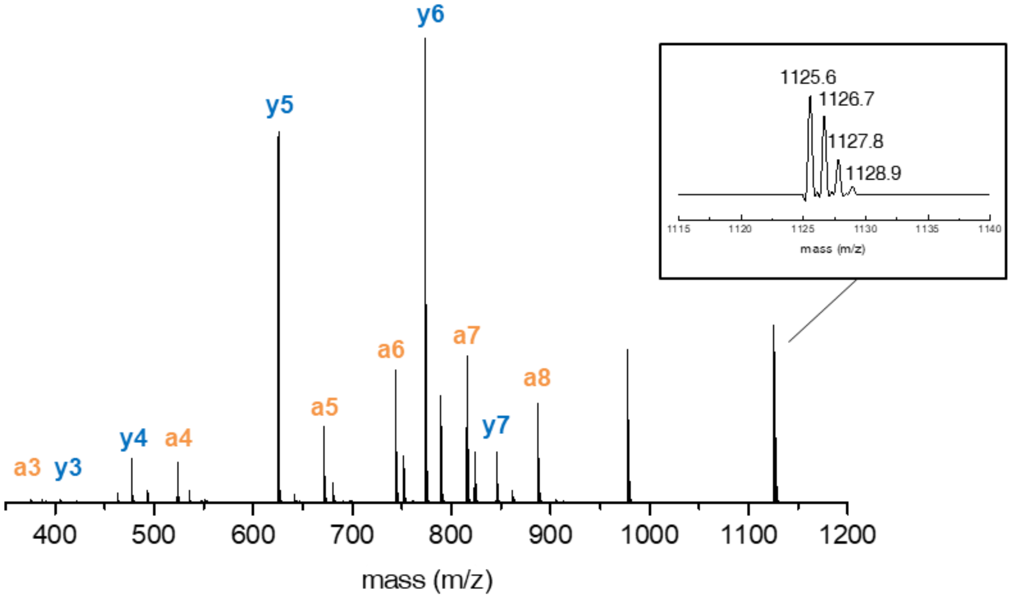

Supplementary Figure 16. MALDI-MS/MS spectrum with peak assignment of PcL (8-bit, N)

Supplementary Table 5. Decoding table of PcL (8-bit, N)

N (PLPPLLLP)									
Si → Bz	Calculated m/z	Found m/z	Difference	Sequence	Bz → Si	Calculated m/z	Found m/z	Difference	Sequence
[M+Na]+	1125.4	1125.6			[M+Na]+	1125.4	1125.6		
y7	845.3	845.5	280.1	Si-P	a8	887.3	887.5	238.1	Bz-P
y6	773.3	773.6	71.9	L	a7	815.3	815.6	71.9	L
y5	625.2	625.5	148.1	P	a6	743.3	743.6	72.0	L
y4	477.2	477.4	148.1	P	a5	671.3	671.6	72.0	L
y3	405.1	405.4	72.0	L	a4	523.2	523.5	148.1	P
y2	333.1	333.3	72.1	L	a3	375.2	375.4	148.1	P
y1	261.1	261.3	72.0	L	a2	303.1	303.3	72.1	L

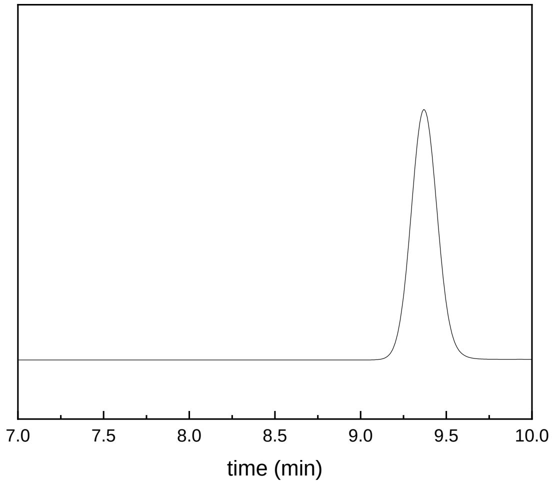

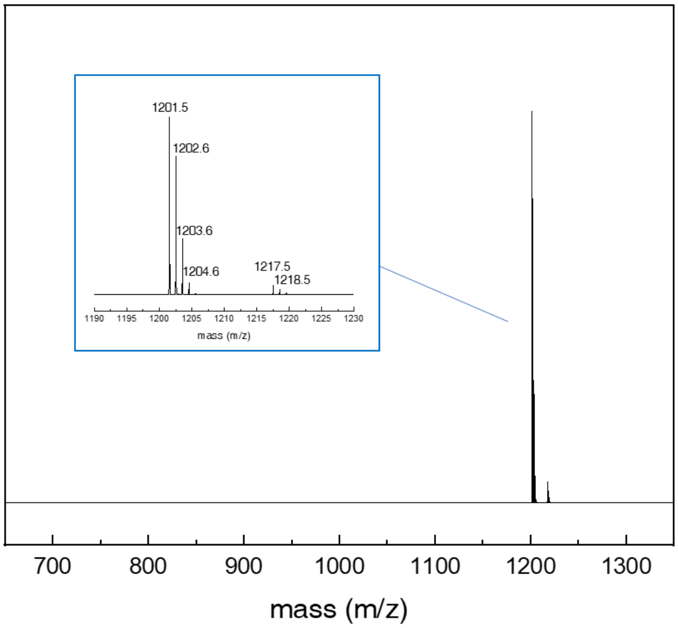

Supplementary Figure 17. GPC and MALDI-TOF MS spectra of PcL (8-bit, C) (theoretical isotopic maximum mass $[M+Na]^+$ = 1201.5 Da).

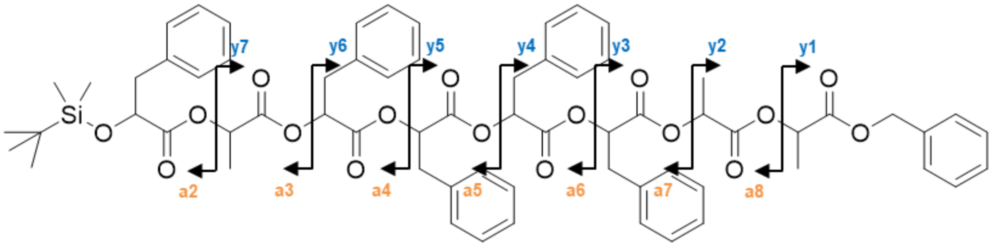

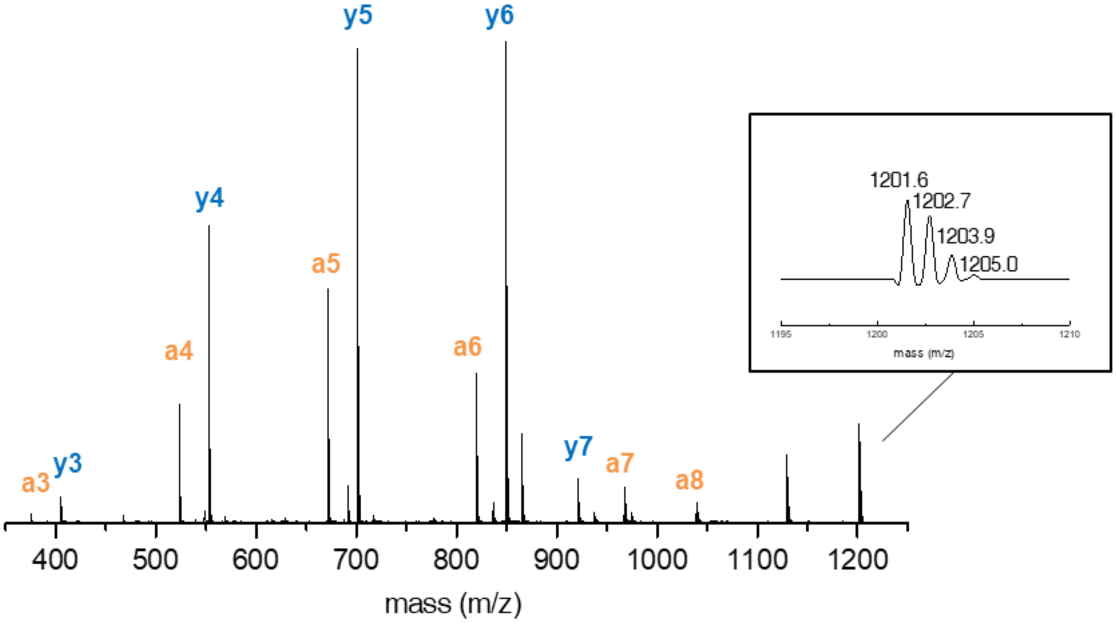

Supplementary Figure 18. MALDI-MS/MS spectrum with peak assignment of PcL (8-bit, C)

Supplementary Table 6. Decoding table of PcL (8-bit, C)

C (PLPPPPLL)									
Si → Bz	Calculated m/z	Found m/z	Difference	Sequence	Bz → Si	Calculated m/z	Found m/z	Difference	Sequence
$[M+Na]^+$	1201.5	1201.6			$[M+Na]^+$	1201.5	1201.6		
y7	921.3	921.5	280.1	Si-P	a8	1039.4	1039.5	162.1	Bz-L
y6	849.3	849.5	72.0	L	a7	967.4	967.5	72.0	L
y5	701.2	701.4	148.1	P	a6	819.3	819.5	148.0	P
y4	553.2	553.4	148.0	P	a5	671.3	671.4	148.1	P
y3	405.1	405.3	148.1	P	a4	523.2	523.4	148.0	P
y2	257.1	257.3	148.0	P	a3	375.2	375.3	148.1	P
y1	185.1	185.2	72.1	L	a2	303.1	303.3	72.0	L

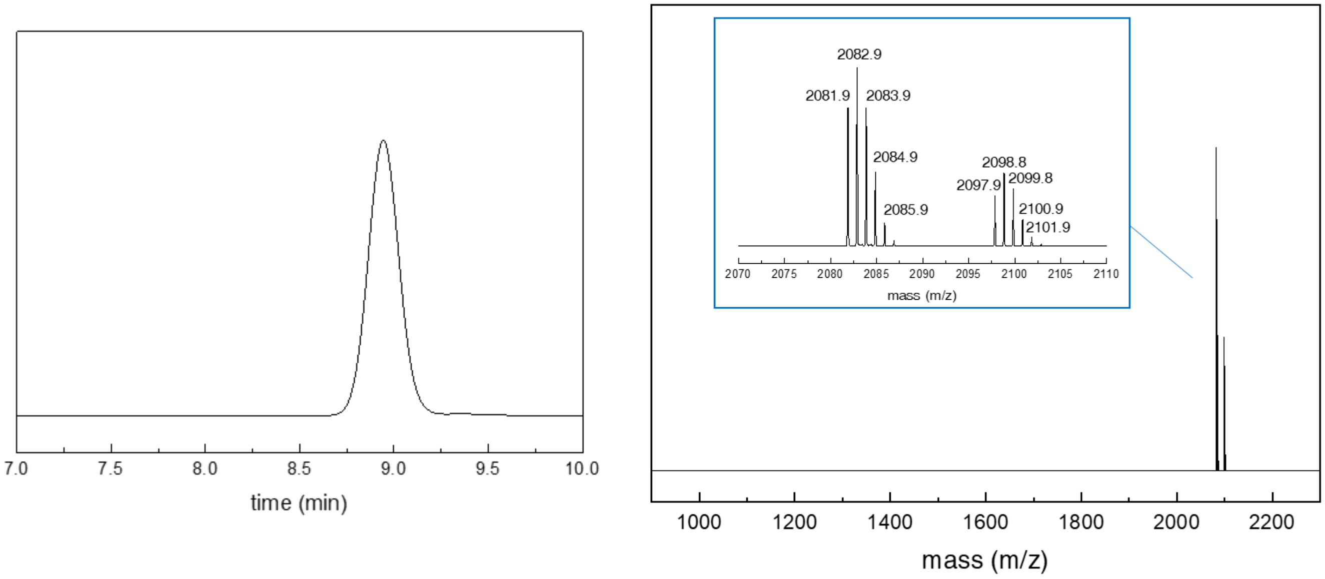

Supplementary Figure 19. GPC and MALDI-TOF MS spectra of PcL (16-bit, SE) (theoretical isotopic maximum mass $[M+Na]^+$ = 2082.8 Da).

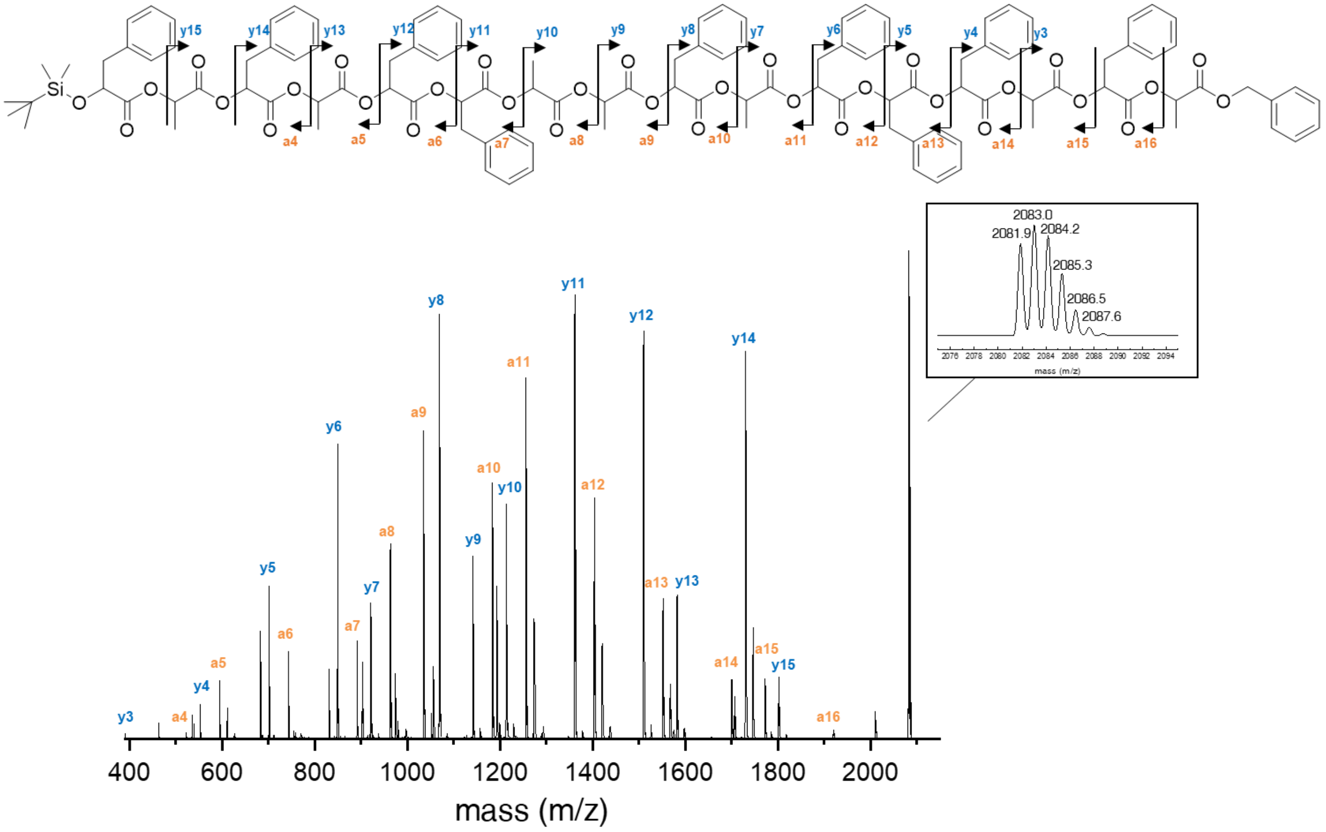

Supplementary Figure 20. MALDI-MS/MS spectrum with peak assignment of PcL (16-bit, SE)

Supplementary Table 7. Decoding table of PcL (16-bit, SE)

SE (PLPLPPLLPLPPPLPL)									
Si → Bz	Calculated m/z	Found m/z	Difference	Sequence	Bz → Si	Calculated m/z	Found m/z	Difference	Sequence
[M+Na]+	2081.8	2083.0			[M+Na]+	2081.8	2083.0		
y15	1801.6	1802.7	280.3	Si-P	a16	1919.7	1920.8	162.2	Bz-L
y14	1729.6	1730.7	72.0	L	a15	1771.6	1772.7	148.1	P
y13	1581.5	1582.7	148.0	P	a14	1699.6	1700.7	72.0	L
y12	1509.5	1510.7	72.0	L	a13	1551.6	1552.7	148.0	P
y11	1361.5	1361.7	149.0	P	a12	1403.5	1403.7	149.0	P
y10	1213.4	1213.6	148.1	P	a11	1255.5	1255.7	148.0	P
y9	1141.4	1141.6	72.0	L	a10	1183.4	1183.7	72.0	L
y8	1069.4	1069.6	72.0	L	a9	1035.4	1035.7	148.0	P
y7	921.3	921.5	148.1	P	a8	963.4	963.6	72.1	L
y6	849.3	849.5	72.0	L	a7	891.3	891.6	72.0	L
y5	701.2	701.4	148.1	P	a6	743.3	743.5	148.1	P
y4	553.2	553.4	148.0	P	a5	595.2	595.5	148.0	P
y3	405.1	405.3	148.1	P	a4	523.2	523.4	72.1	L

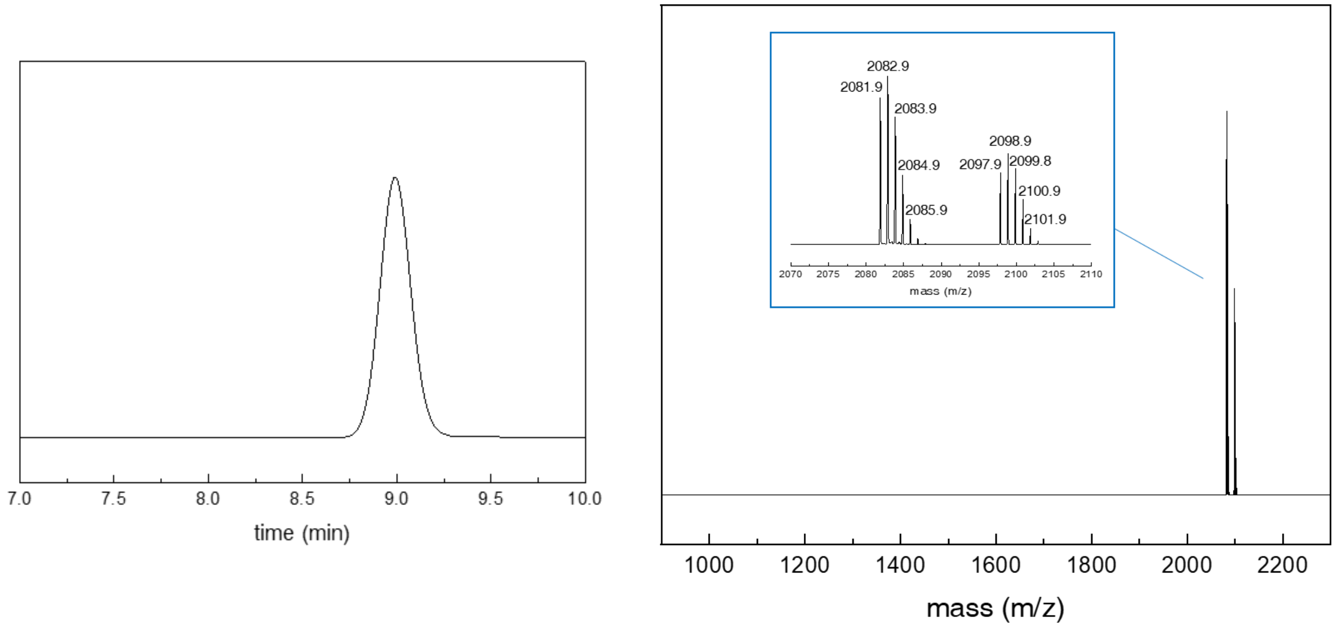

Supplementary Figure 21. GPC and MALDI-TOF MS spectra of PcL (16-bit, QU) (theoretical isotopic maximum mass $[M+Na]^+$ = 2082.8 Da).

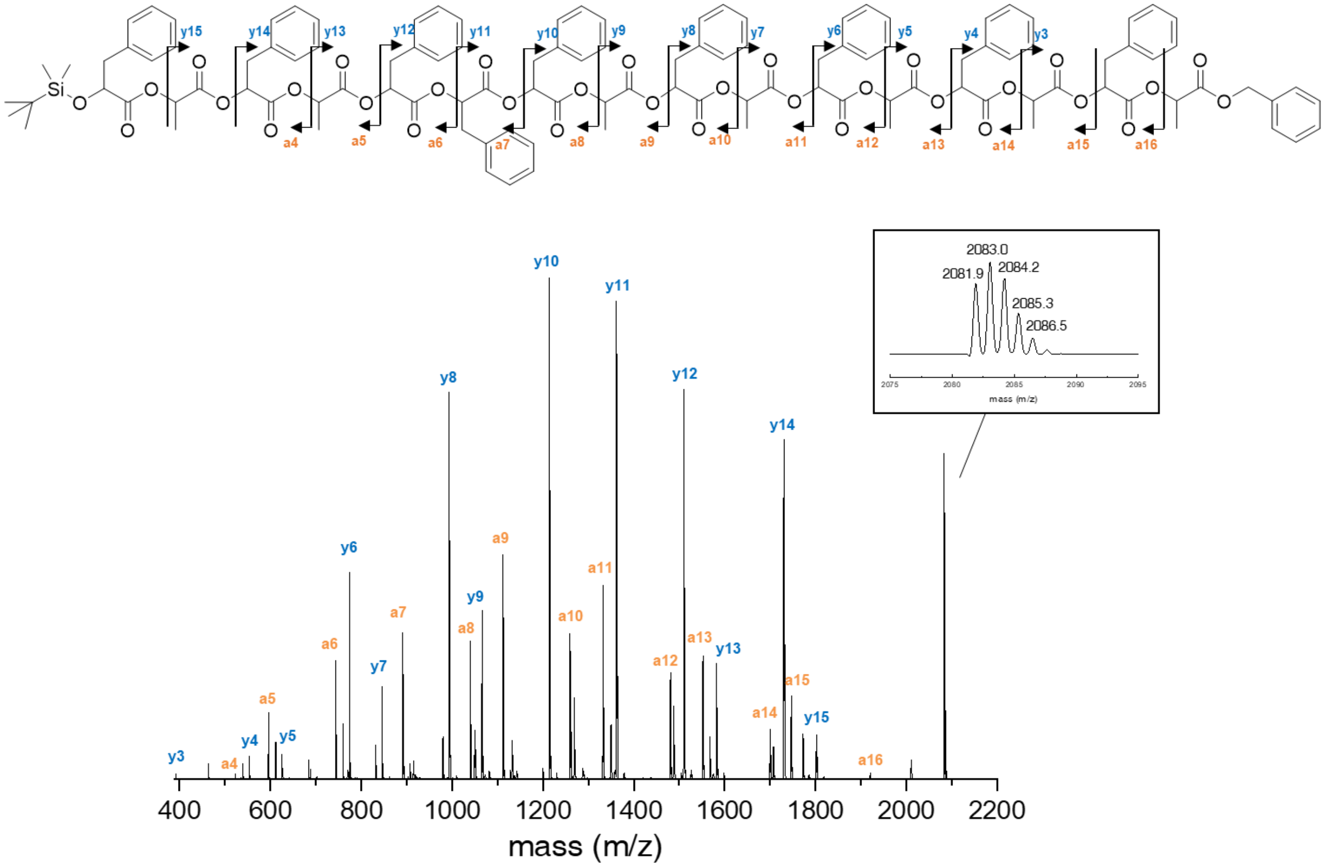

Supplementary Figure 22. MALDI-MS/MS spectrum with peak assignment of PcL (16-bit, QU)

Supplementary Table 8. Decoding table of PcL (16-bit, QU)

QU (PLPLPPPLPLPLPLPL)									
Si → Bz	Calculated m/z	Found m/z	Difference	Sequence	Bz → Si	Calculated m/z	Found m/z	Difference	Sequence
$[M+Na]^+$	2081.8	2083.1			$[M+Na]^+$	2081.8	2083.1		
y15	1801.6	1802.6	280.5	Si-P	a16	1919.7	1920.7	162.4	Bz-L
y14	1729.6	1730.6	72.0	L	a15	1771.6	1772.6	148.1	P
y13	1581.5	1582.6	148.0	P	a14	1699.6	1700.6	72.0	L
y12	1509.5	1510.6	72.0	L	a13	1551.6	1552.6	148.0	P
y11	1361.5	1361.6	149.0	P	a12	1479.5	1480.6	72.0	L
y10	1213.4	1213.6	148.0	P	a11	1331.5	1331.6	149.0	P
y9	1065.4	1065.6	148.0	P	a10	1259.5	1259.7	71.9	L
y8	993.3	993.5	72.1	L	a9	1111.4	1111.6	148.1	P
y7	845.3	845.5	148.0	P	a8	1039.4	1039.6	72.0	L
y6	773.3	773.5	72.0	L	a7	891.3	891.5	148.1	P
y5	625.2	625.4	148.1	P	a6	743.3	743.5	148.0	P
y4	553.2	553.4	72.0	L	a5	595.2	595.4	148.1	P
y3	405.1	405.4	148.0	P	a4	523.2	523.4	72.0	L

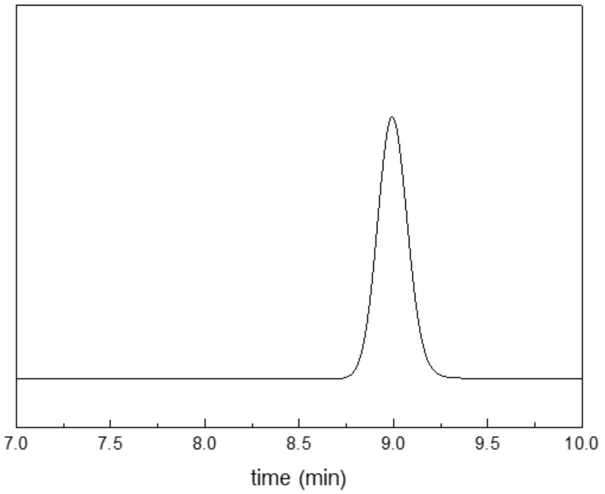

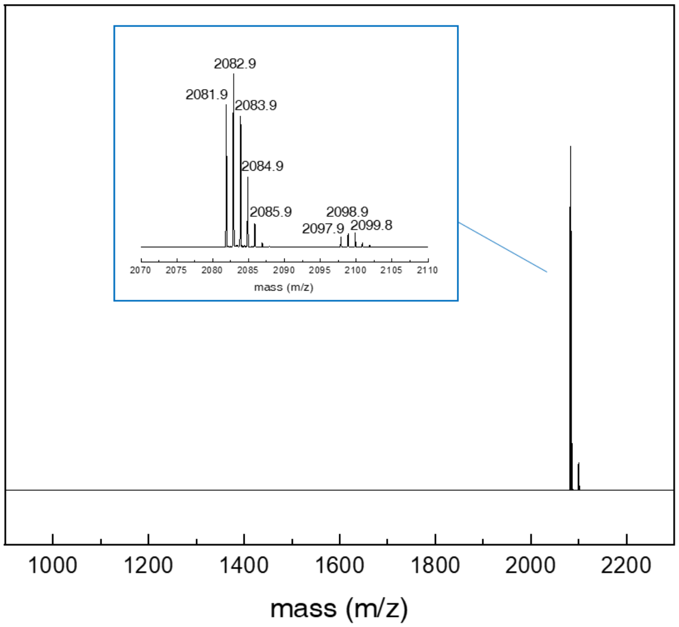

Supplementary Figure 23. GPC and MALDI-TOF MS spectra of PcL (16-bit, EN) (theoretical isotopic maximum mass $[M+Na]^+$ = 2082.8 Da).

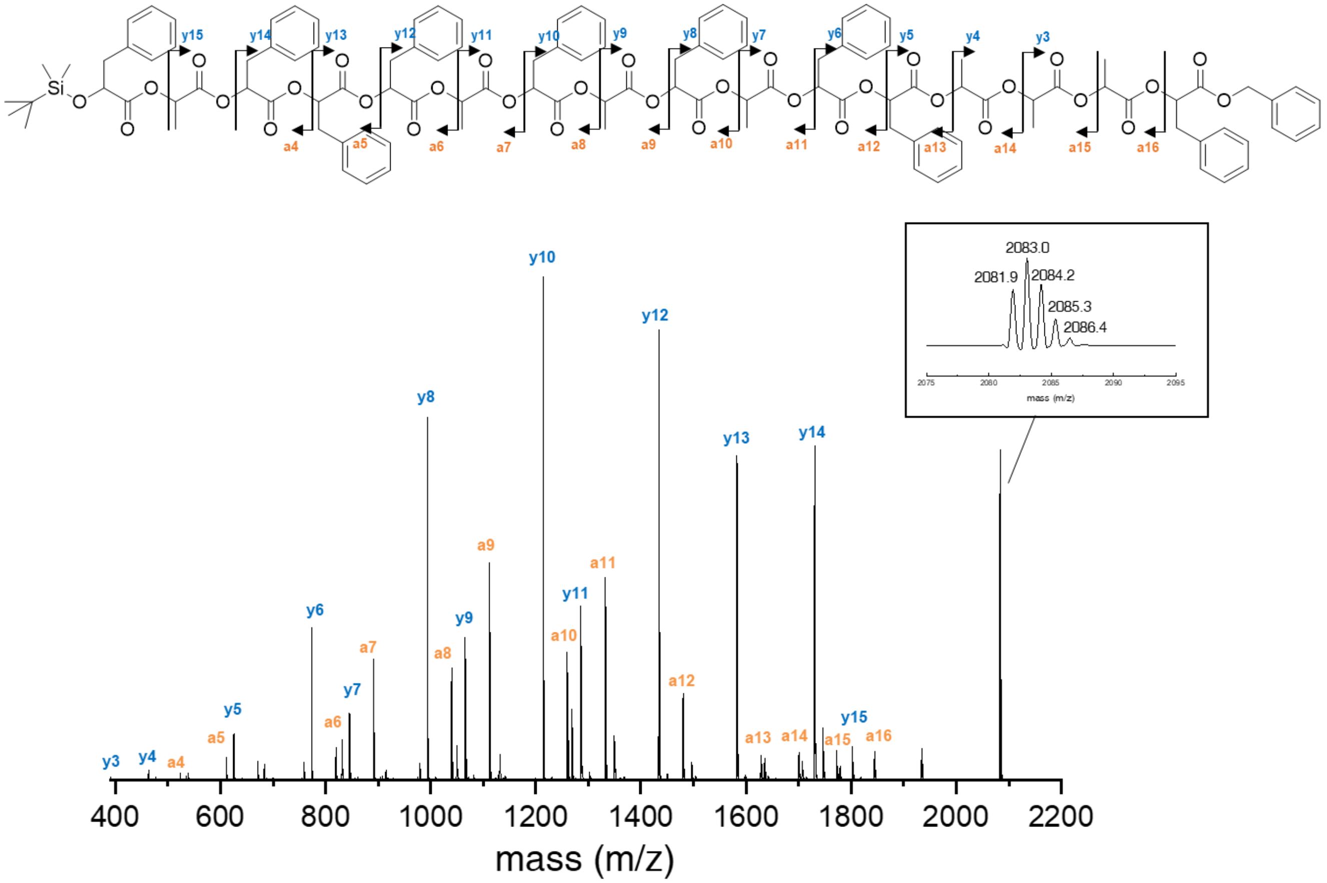

Supplementary Figure 24. MALDI-MS/MS spectrum with peak assignment of PcL (16-bit, EN)

Supplementary Table 9. Decoding table of PcL (16-bit, EN)

EN (PLPPPLPLPLPPLLLP)									
Si → Bz	Calculated m/z	Found m/z	Difference	Sequence	Bz → Si	Calculated m/z	Found m/z	Difference	Sequence
[M+Na]$^+$	2081.8	2083.0			[M+Na]$^+$	2081.8	2083.0		
y15	1801.6	1802.8	280.2	Si-P	a16	1843.7	1844.8	238.2	Bz-P
y14	1729.6	1730.8	72.0	L	a15	1771.6	1772.8	72.0	L
y13	1581.5	1582.8	148.0	P	a14	1699.6	1700.8	72.0	L
y12	1433.5	1433.8	149.0	P	a13	1627.6	1628.8	72.0	L
y11	1285.4	1285.7	148.1	P	a12	1479.5	1480.8	148.0	P
y10	1213.4	1213.7	72.0	L	a11	1331.5	1331.8	149.0	P
y9	1065.4	1065.7	148.0	P	a10	1259.5	1259.8	72.0	L
y8	993.3	993.6	72.1	L	a9	1111.4	1111.7	148.1	P
y7	845.3	845.6	148.0	P	a8	1039.4	1039.7	72.0	L
y6	773.3	773.6	72.0	L	a7	891.3	891.6	148.1	P
y5	625.2	625.5	148.1	P	a6	819.3	819.6	72.0	L
y4	477.2	477.3	148.2	P	a5	671.3	671.5	148.1	P
y3	405.1	405.3	72.0	L	a4	523.2	523.5	148.0	P

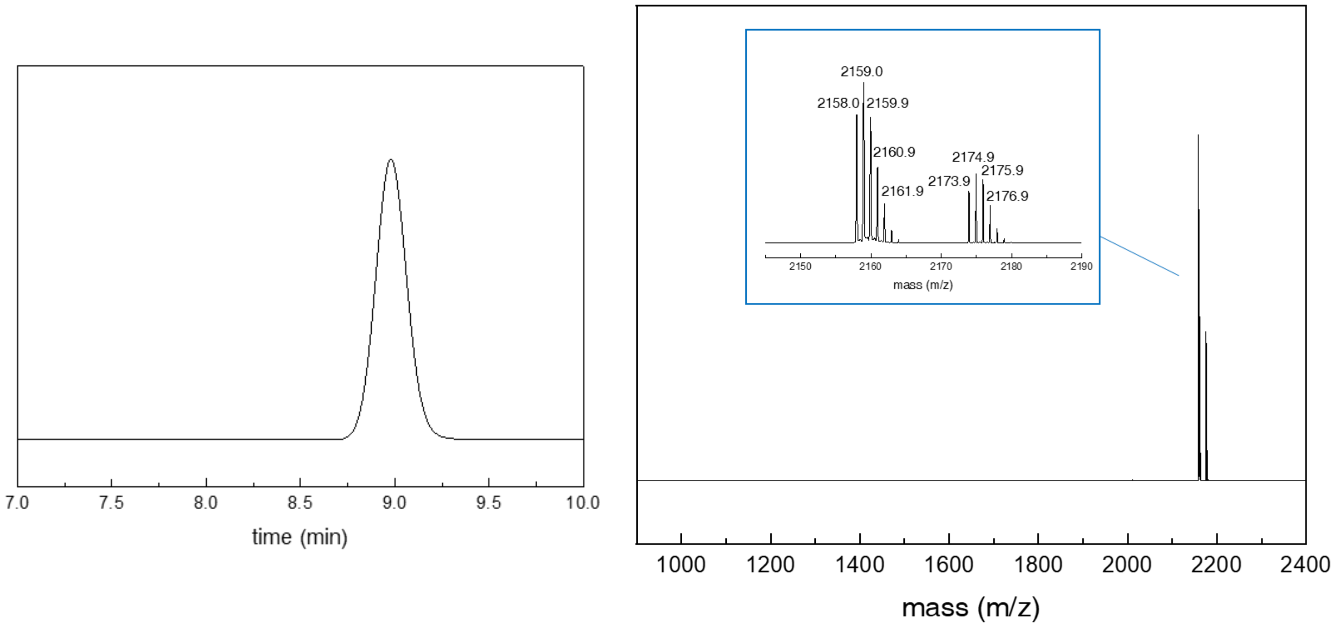

Supplementary Figure 25. GPC and MALDI-TOF MS spectra of PcL (16-bit, CE) (theoretical isotopic maximum mass $[M+Na]^{+}$ = 2158.8 Da).

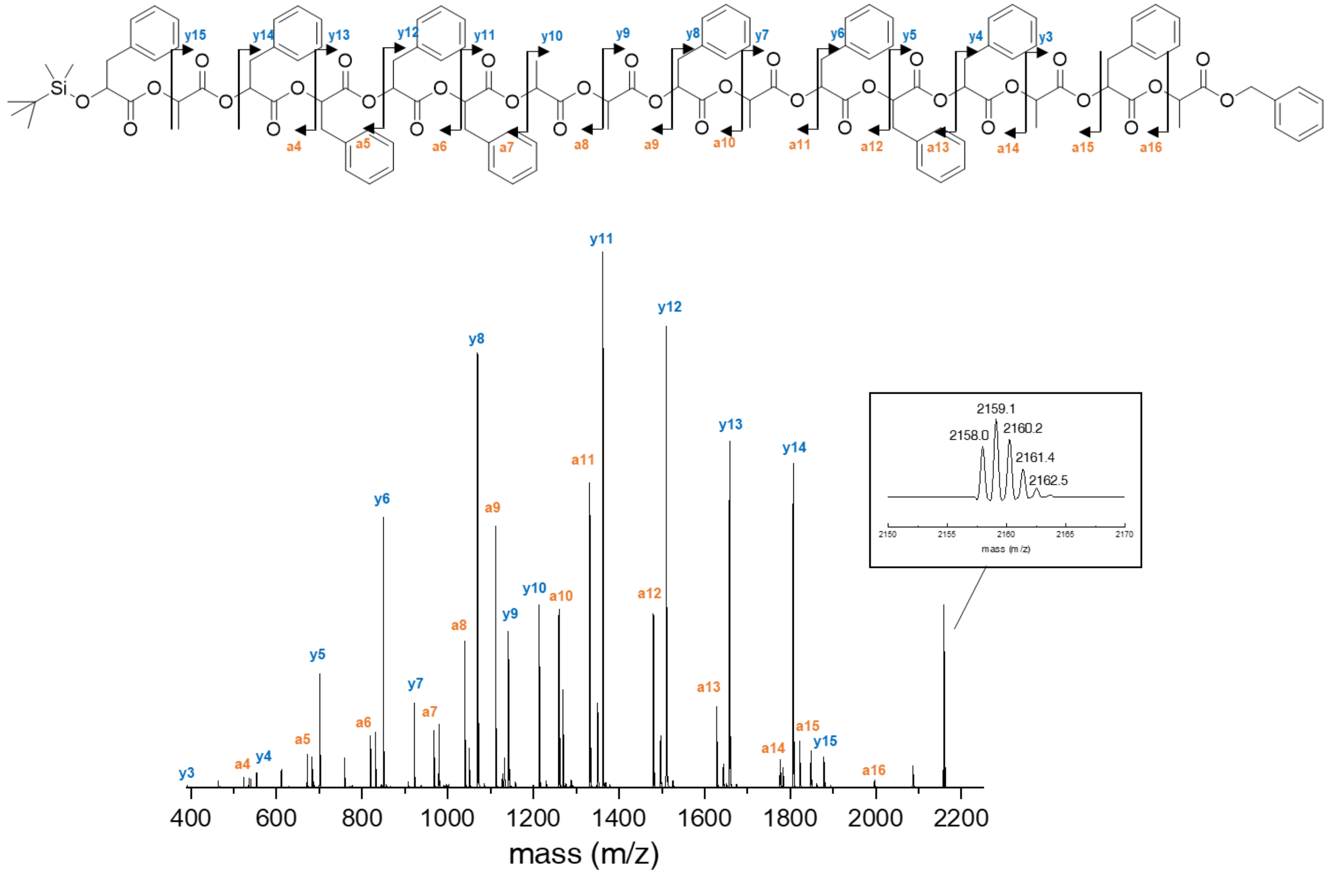

Supplementary Figure 26. MALDI-MS/MS spectrum with peak assignment of PcL (16-bit, CE)

Supplementary Table 10. Decoding table of PcL (16-bit, CE)

CE (PLPPPPLLPLPPPLPL)									
Si → Bz	Calculated m/z	Found m/z	Difference	Sequence	Bz → Si	Calculated m/z	Found m/z	Difference	Sequence
[M+Na]$^{+}$	2157.8	2159.1			[M+Na]$^{+}$	2157.8	2159.1		
y15	1877.6	1878.8	280.3	Si-P	a16	1995.7	1996.9	162.2	Bz-L
y14	1805.6	1806.8	72.0	L	a15	1847.7	1848.8	148.1	P
y13	1657.6	1658.8	148.0	P	a14	1775.6	1776.8	72.0	L
y12	1509.5	1510.8	148.0	P	a13	1627.6	1628.8	148.0	P
y11	1361.5	1361.8	149.0	P	a12	1479.5	1479.8	149.0	P
y10	1213.4	1213.7	148.1	P	a11	1331.5	1331.8	148.0	P
y9	1141.4	1141.7	72.0	L	a10	1259.5	1259.8	72.0	L
y8	1069.4	1069.7	72.0	L	a9	1111.4	1111.7	148.1	P
y7	921.3	921.6	148.1	P	a8	1039.4	1039.7	72.0	L
y6	849.3	849.6	72.0	L	a7	967.4	967.7	72.0	L
y5	701.2	701.5	148.1	P	a6	819.3	819.6	148.1	P
y4	553.2	553.4	148.1	P	a5	671.3	671.5	148.1	P
y3	405.1	405.2	148.2	P	a4	523.2	523.5	148.0	P

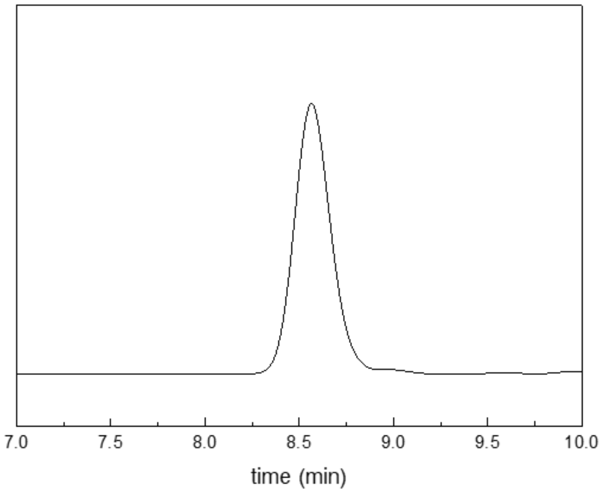

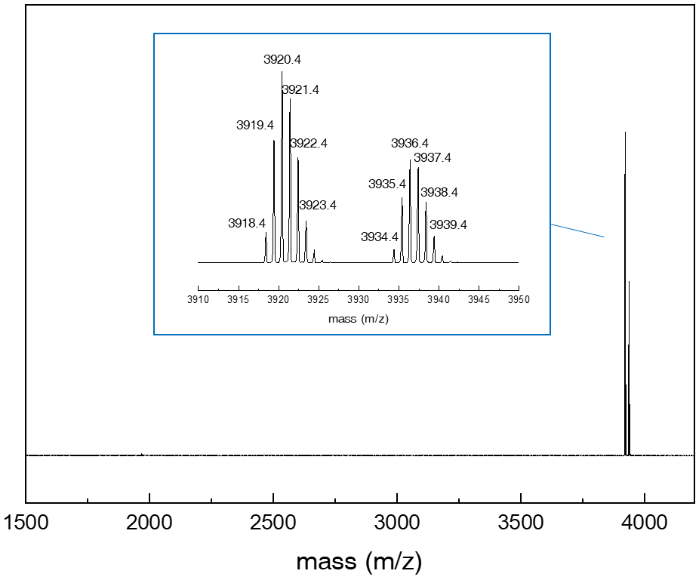

Supplementary Figure 27. GPC and MALDI-TOF MS spectra of PcL (32-bit, SEQU) (theoretical isotopic maximum mass $[M+Na]^+$ = 3920.4 Da).

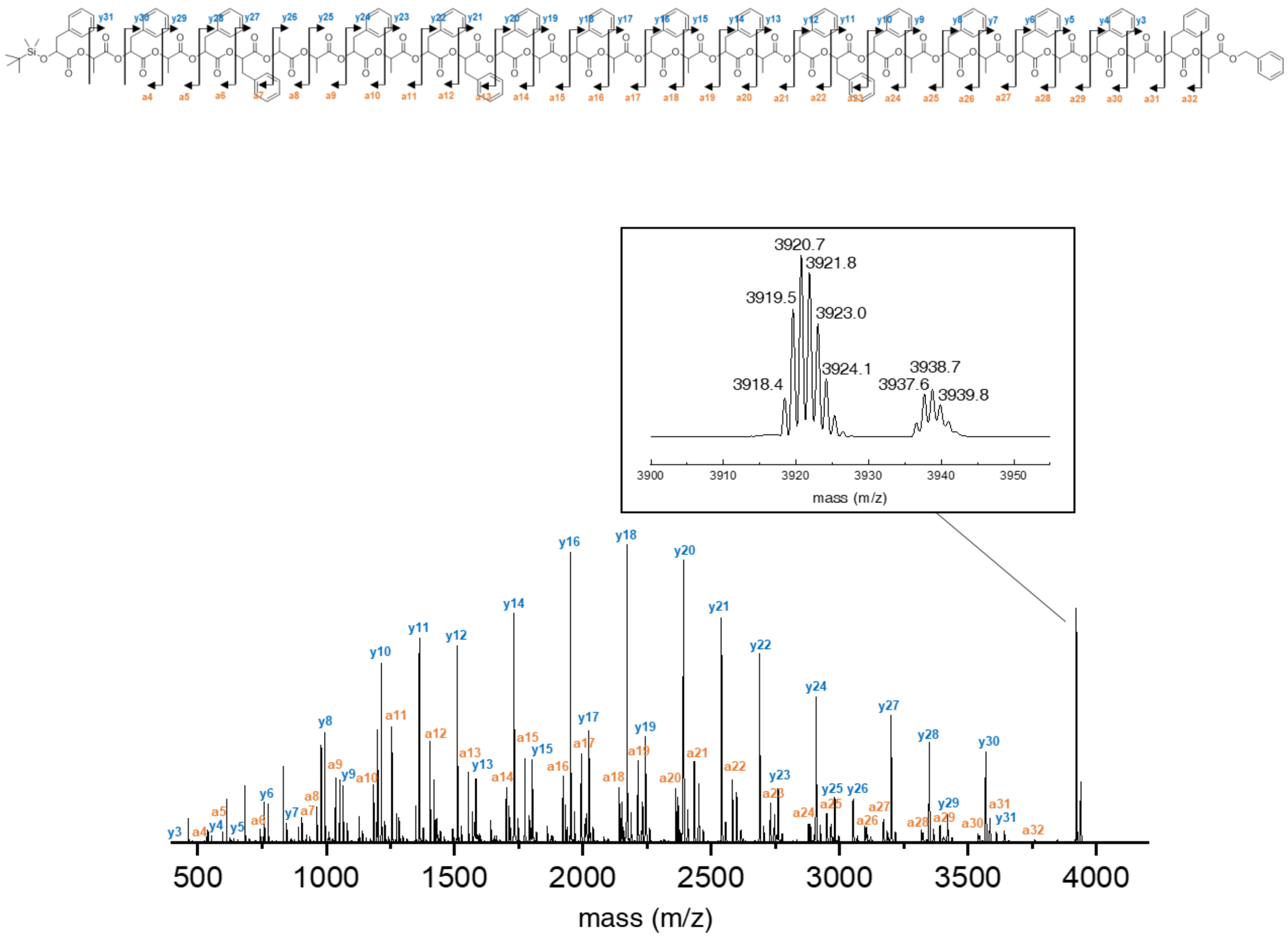

Supplementary Figure 28. MALDI-MS/MS spectrum with peak assignment of PcL (32-bit, SEQU)

Supplementary Table 11. Decoding table of PcL (32-bit, SEQU)

SEQU (PLPLPPLLPLPPPLPLPLPLPPPLPLPLPLPL)									
Si → Bz	Calculated m/z	Found m/z	Difference	Sequence	Bz → Si	Calculated m/z	Found m/z	Difference	Sequence
[M+Na]+	3918.4	3920.7			[M+Na]+	3918.4	3920.7		
y31	3638.2	3640.6	280.1	Si-P	a32	3756.3	3758.7	162.0	Bz-L
y30	3566.2	3568.6	72.0	L	a31	3608.2	3610.7	148.0	P
y29	3418.2	3420.5	148.1	P	a30	3536.2	3538.6	72.1	L
y28	3346.1	3348.5	72.0	L	a29	3388.2	3390.6	148.0	P
y27	3198.1	3200.4	148.1	P	a28	3316.2	3318.6	72.0	L
y26	3050.0	3052.5	147.9	P	a27	3168.1	3170.4	148.2	P
y25	2978.0	2980.5	72.0	L	a26	3096.1	3098.6	71.8	L
y24	2906.0	2907.5	73.0	L	a25	2948.0	2949.5	149.1	P
y23	2757.9	2759.4	148.1	P	a24	2876.0	2877.5	72.0	L
y22	2685.9	2687.5	71.9	L	a23	2728.0	2729.5	148.0	P
y21	2537.9	2539.5	148.0	P	a22	2579.9	2581.5	148.0	P
y20	2389.8	2391.5	148.0	P	a21	2431.8	2433.5	148.0	P
y19	2241.8	2243.4	148.1	P	a20	2359.8	2361.5	72.0	L
y18	2169.7	2171.5	71.9	L	a19	2211.8	2213.5	148.0	P
y17	2021.7	2023.4	148.1	P	a18	2139.8	2141.5	72.0	L
y16	1949.7	1951.4	72.0	L	a17	1991.7	1993.5	148.0	P
y15	1801.6	1803.4	148.0	P	a16	1919.7	1921.5	72.0	L
y14	1729.6	1731.4	72.0	L	a15	1771.6	1773.4	148.1	P
y13	1581.5	1583.3	148.1	P	a14	1699.6	1701.4	72.0	L
y12	1509.5	1510.3	73.0	L	a13	1551.6	1553.3	148.1	P
y11	1361.5	1362.2	148.1	P	a12	1403.5	1404.3	149.0	P
y10	1213.4	1214.2	148.0	P	a11	1255.4	1256.2	148.1	P
y9	1065.4	1066	148.2	P	a10	1183.4	1184.2	72.0	L
y8	993.3	994.0	72.0	L	a9	1035.4	1036.1	148.1	P
y7	845.3	846.0	148.0	P	a8	963.4	964.1	72.0	L
y6	773.3	774.0	72.0	L	a7	891.3	892.0	72.1	L
y5	625.2	625.8	148.2	P	a6	743.3	743.9	148.1	P
y4	553.2	553.8	72.0	L	a5	595.2	595.8	148.1	P
y3	405.1	405.6	148.2	P	a4	523.2	523.8	72.0	L

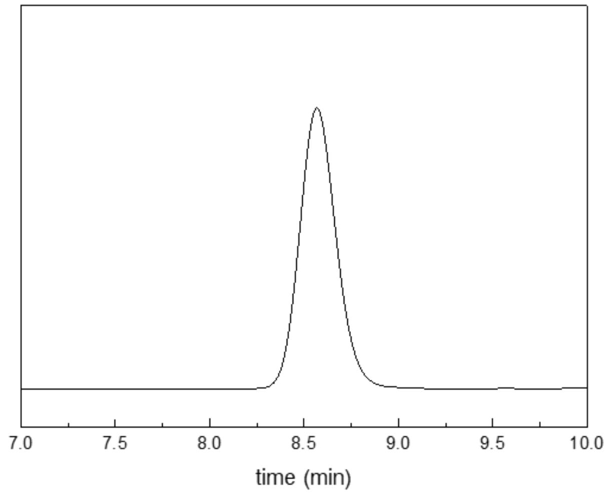

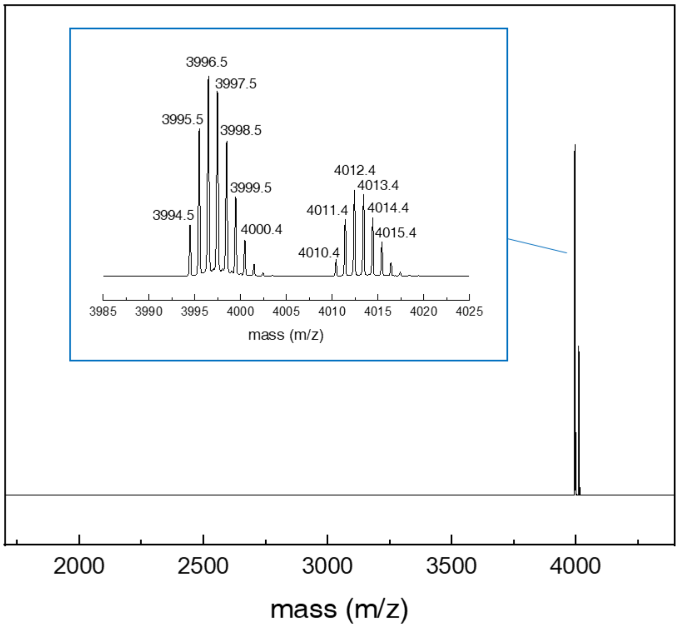

Supplementary Figure 29. GPC and MALDI-TOF MS spectra of PcL (32-bit, ENCE) (theoretical isotopic maximum mass $[M+Na]^+$ = 3996.4 Da).

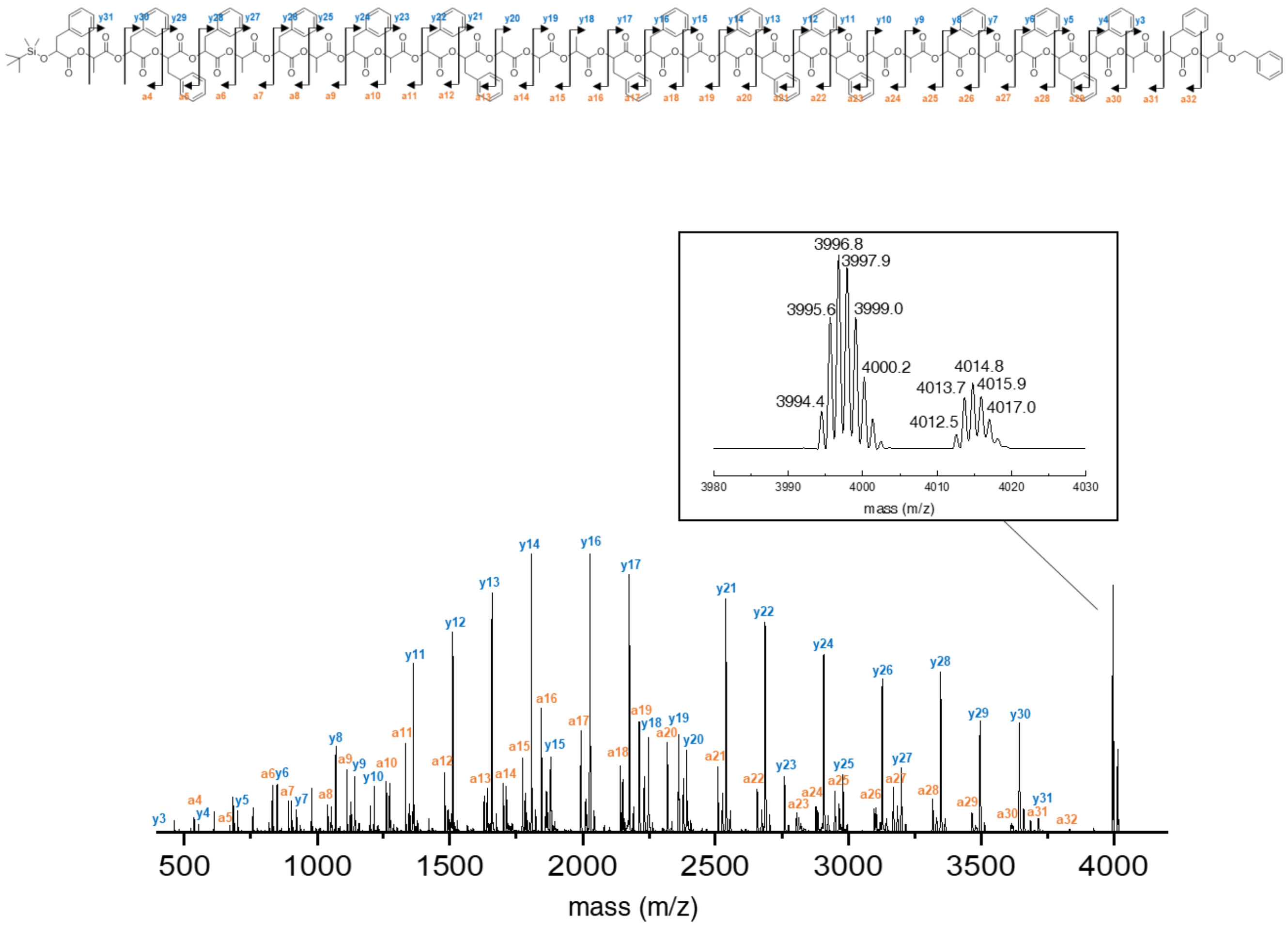

Supplementary Figure 30. MALDI-MS/MS spectrum with peak assignment of PcL (32-bit, ENCE)

Supplementary Table 12. Decoding table of PcL (32-bit, ENCE)

ENCE (PLPPPLPLPLPPLLLPPLPPPPLLPLPPPLPL)

Si → Bz	Calculated m/z	Found m/z	Difference	Sequence	Bz → Si	Calculated m/z	Found m/z	Difference	Sequence
[M+Na]$^{+}$	3994.4	3996.8			[M+Na]$^{+}$	3994.4	3996.8		
y31	3714.3	3716.7	280.1	Si-P	a32	3832.3	3834.4	162.4	Bz-L
y30	3642.2	3644.6	72.1	L	a31	3684.3	3686.5	147.9	P
y29	3494.2	3496.6	148.0	P	a30	3612.3	3614.6	71.9	L
y28	3346.1	3348.5	148.1	P	a29	3464.2	3466.4	148.2	P
y27	3198.1	3200.2	148.3	P	a28	3316.2	3318.6	147.8	P
y26	3126.1	3128.4	71.8	L	a27	3168.1	3170.5	148.1	P
y25	2978.0	2980.5	147.9	P	a26	3096.1	3097.5	73.0	L
y24	2906.0	2908.4	72.1	L	a25	2948.0	2950.4	147.1	P
y23	2757.9	2759.4	149.0	P	a24	2876.0	2877.5	72.9	L
y22	2685.9	2687.5	71.9	L	a23	2804.0	2805.6	71.9	L
y21	2537.9	2539.5	148.0	P	a22	2655.9	2657.5	148.1	P
y20	2389.8	2391.4	148.1	P	a21	2507.9	2509.5	148.0	P
y19	2317.8	2319.4	72.0	L	a20	2359.8	2361.5	148.0	P
y18	2245.8	2247.4	72.0	L	a19	2211.8	2213.5	148.0	P
y17	2173.8	2175.4	72.0	L	a18	2139.8	2141.4	72.1	L
y16	2025.7	2027.4	148.0	P	a17	1991.7	1993.4	148.0	P
y15	1877.6	1879.4	148.0	P	a16	1843.6	1845.4	148.0	P
y14	1805.6	1807.3	72.1	L	a15	1771.6	1773.4	72.0	L
y13	1657.6	1659.3	148.0	P	a14	1699.6	1701.4	72.0	L
y12	1509.5	1510.3	149.0	P	a13	1627.6	1629.3	72.1	L
y11	1361.5	1362.2	148.1	P	a12	1479.5	1481.3	148.0	P
y10	1213.4	1214.2	148.0	P	a11	1331.5	1332.3	149.0	P
y9	1141.4	1142.1	72.1	L	a10	1259.5	1260.3	72.0	L
y8	1069.4	1070.1	72.0	L	a9	1111.4	1112.2	148.1	P
y7	921.3	922.0	148.1	P	a8	1039.4	1040.1	72.1	L
y6	849.3	849.9	72.1	L	a7	891.3	892.0	148.1	P
y5	701.2	701.8	148.1	P	a6	819.3	820.0	72.0	L
y4	553.2	553.7	148.1	P	a5	671.3	671.8	148.2	P
y3	405.1	405.5	148.2	P	a4	523.2	523.7	148.1	P

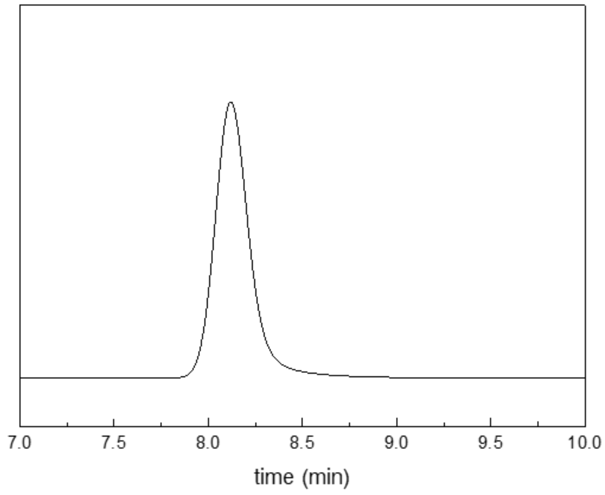

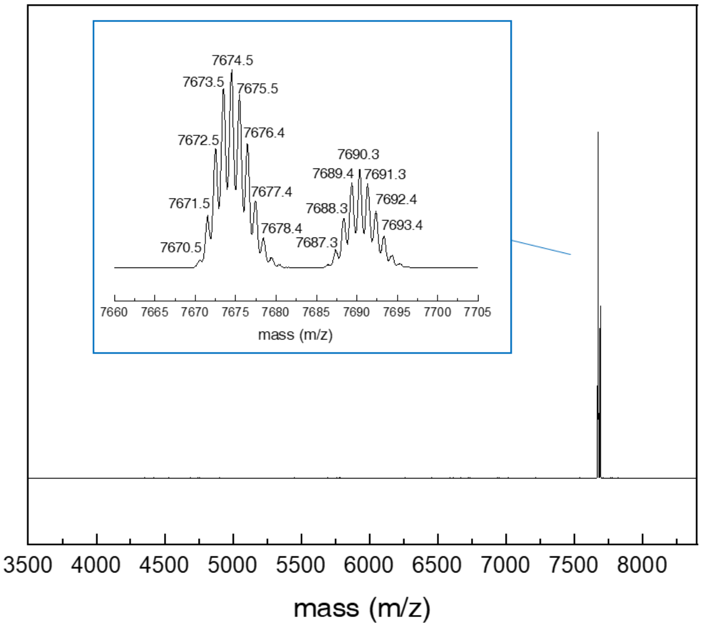

Supplementary Figure 31. GPC and MALDI-TOF MS spectra of PcL (64-bit, SEQUENCE) (theoretical isotopic maximum mass $[M+Na]^+$ = 7672.6 Da).

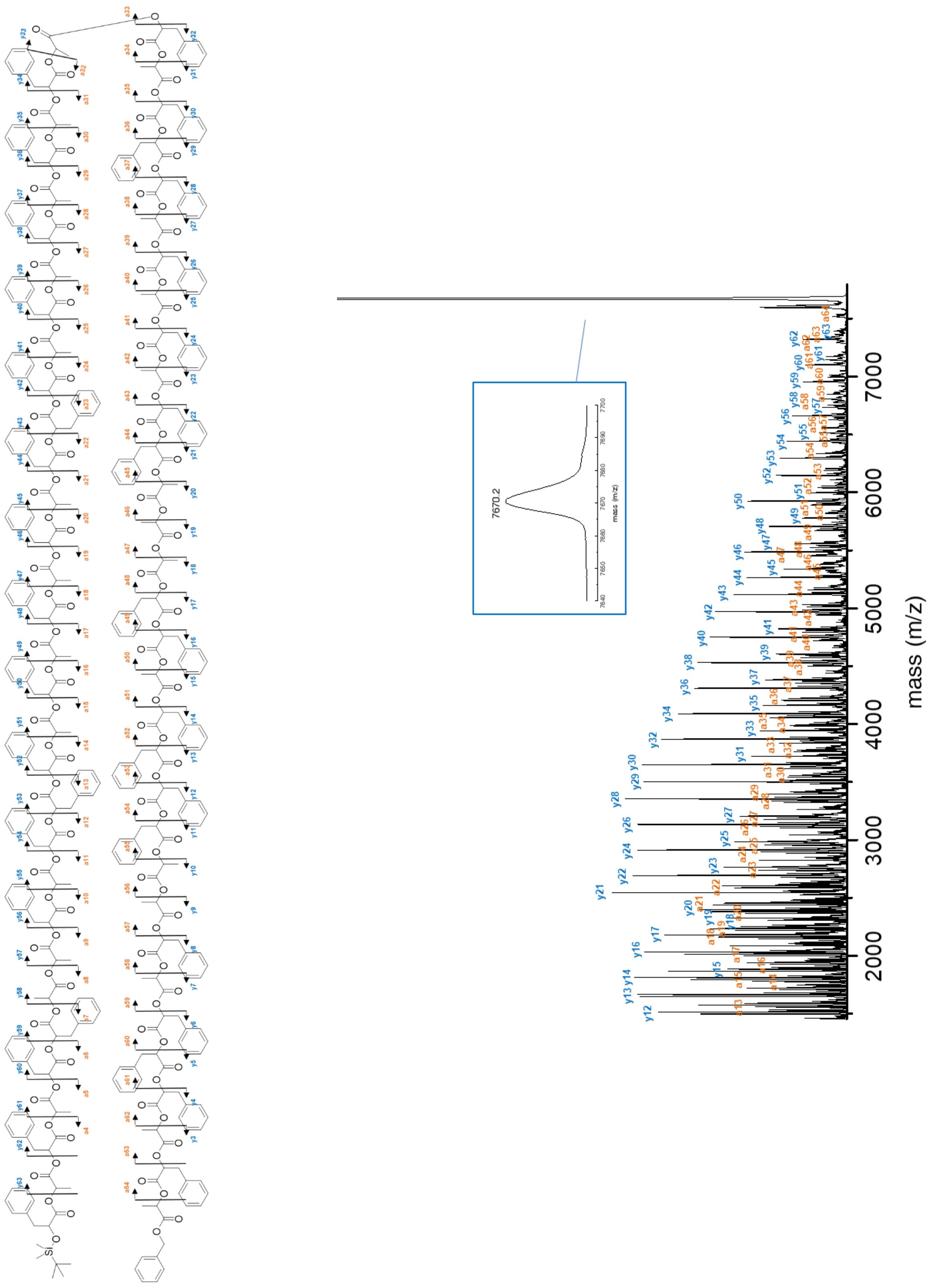

Supplementary Figure 32. MALDI-MS/MS spectrum with peak assignment of PcL (64-bit, SEQUENCE, PLPLPPLLPLPPPLPLPLPLPPPLPLPLPLPLPPPLPLPLPPLLLPPLPPPPLLPLPPPLPL)

Supplementary Table 13. Decoding table of PcL (64-bit, SEQUENCE)

Si → Bz	Calculated m/z	Found m/z	Difference	Sequence	Bz → Si	Calculated m/z	Found m/z	Difference	Sequence
$[M+Na]^+$	7667.6	7670.2			$[M+Na]^+$	7667.6	7670.2		
y63	7387.5	7394.6	275.6	Si-P	a64	7505.6	7514.3	155.9	Bz-L
y62	7315.5	7322.6	72.0	L	a63	7357.5	7365.3	149.0	P
y61	7167.4	7174.2	148.4	P	a62	7285.5	7294.0	71.3	L
y60	7095.4	7102.1	72.1	L	a61	7137.4	7145.7	148.3	P
y59	6947.4	6955.5	146.6	P	a60	6989.4	6997.2	148.5	P
y58	6799.3	6806.4	149.1	P	a59	6841.3	6849.3	147.9	P
y57	6727.3	6734.3	72.1	L	a58	6769.3	6776.1	73.2	L
y56	6655.3	6662.3	72.0	L	a57	6621.3	6627.5	148.6	P
y55	6507.2	6515.2	147.1	P	a56	6549.2	6556.9	70.6	L
y54	6435.2	6442.5	72.7	L	a55	6477.2	6484.8	72.1	L
y53	6287.1	6295.5	147.0	P	a54	6329.2	6336.1	148.7	P
y52	6139.1	6146.5	149.0	P	a53	6181.1	6189.1	147.0	P
y51	5991.0	5998.5	148.0	P	a52	6033.1	6040.4	148.7	P
y50	5919.0	5926.0	72.5	L	a51	5885.0	5893.3	147.1	P
y49	5771.0	5778.4	147.6	P	a50	5813.0	5820.6	72.7	L
y48	5698.9	5706.6	71.8	L	a49	5664.9	5672.1	148.5	P
y47	5550.9	5558.8	147.8	P	a48	5516.9	5524.6	147.5	P
y46	5478.9	5486.3	72.5	L	a47	5444.9	5453.1	71.5	L
y45	5330.8	5339.3	147.0	P	a46	5372.8	5380	73.1	L
y44	5259.8	5266.0	73.3	L	a45	5300.8	5308.8	71.2	L
y43	5110.7	5118.8	147.2	P	a44	5152.8	5160.8	148.0	P
y42	4962.7	4970.5	148.3	P	a43	5004.7	5012.6	148.2	P
y41	4814.6	4822.7	147.8	P	a42	4932.7	4940.8	71.8	L
y40	4742.6	4750.3	72.4	L	a41	4784.6	4792.5	148.3	P
y39	4594.6	4602.6	147.7	P	a40	4712.6	4720.5	72.0	L
y38	4522.5	4530.3	72.3	L	a39	4564.6	4573.1	147.4	P
y37	4374.5	4382.9	147.4	P	a38	4492.6	4501.0	72.1	L
y36	4302.5	4310.2	72.7	L	a37	4344.5	4352.1	148.9	P
y35	4154.4	4162.7	147.5	P	a36	4196.4	4204.6	147.5	P
y34	4082.4	4090.8	71.9	L	a35	4048.4	4056.5	148.1	P
y33	3934.3	3942.3	148.5	P	a34	3976.4	3984.5	72.0	L
y32	3862.3	3870.0	72.3	L	a33	3828.3	3836.1	148.4	P

y31	3714.3	3722.4	147.6	P	a32	3756.3	3764.3	71.8	L
y30	3642.2	3649.6	72.8	L	a31	3608.2	3615.9	148.4	P
y29	3494.2	3501.5	148.1	P	a30	3536.2	3544.3	71.6	L
y28	3346.1	3353.6	147.9	P	a29	3388.2	3396.3	148.0	P
y27	3198.1	3205.5	148.1	P	a28	3316.2	3323.8	72.5	L
y26	3126.1	3133.4	72.1	L	a27	3168.1	3176.2	147.6	P
y25	2978.0	2985.2	148.2	P	a26	3096.1	3103.4	72.8	L
y24	2906.0	2913.4	71.8	L	a25	2948.0	2955.4	148.0	P
y23	2757.9	2765.5	147.9	P	a24	2876.0	2883.8	71.6	L
y22	2685.9	2693.1	72.4	L	a23	2728.0	2735.4	148.4	P
y21	2537.9	2545.2	147.9	P	a22	2579.9	2587.2	148.2	P
y20	2389.8	2396.8	148.4	P	a21	2430.8	2439.1	148.1	P
y19	2317.8	2324.6	72.2	L	a20	2359.8	2367.0	72.1	L
y18	2245.8	2252.3	72.3	L	a19	2211.8	2218.1	148.9	P
y17	2173.8	2180.4	71.9	L	a18	2139.8	2146.3	71.8	L
y16	2025.7	2031.9	148.5	P	a17	1991.7	1997.9	148.4	P
y15	1877.6	1883.6	148.3	P	a16	1919.7	1926.0	71.9	L
y14	1805.6	1811.8	71.8	L	a15	1771.6	1777.8	148.2	P
y13	1657.6	1663.4	148.4	P	a14	1699.6	1705.6	72.2	L
y12	1509.5	1515.2	148.2	P	a13	1551.6	1557.4	148.2	P
y11	1361.5	1367.0	148.2	P	a12	1403.5	1409.1	148.3	P
y10	1213.4	1218.4	148.6	P	a11	1255.4	1260.5	148.6	P
y9	1141.4	1145.8	72.6	L	a10	1183.4	1188.3	72.2	L
y8	1069.4	1074.1	71.7	L	a9	1035.4	1039.9	148.4	P
y7	921.3	925.5	148.6	P	a8	963.4	967.7	72.2	L
y6	849.3	853.3	72.2	L	a7	891.3	895.5	72.2	L
y5	701.2	704.7	148.6	P	a6	743.3	746.9	148.6	P
y4	553.2	555.9	148.8	P	a5	595.2	598.3	148.6	P
y3	405.1	406.5	149.4	P	a4	523.2	525.7	72.6	L

MALDI-TOF sequencing of PcL after hydrolysis

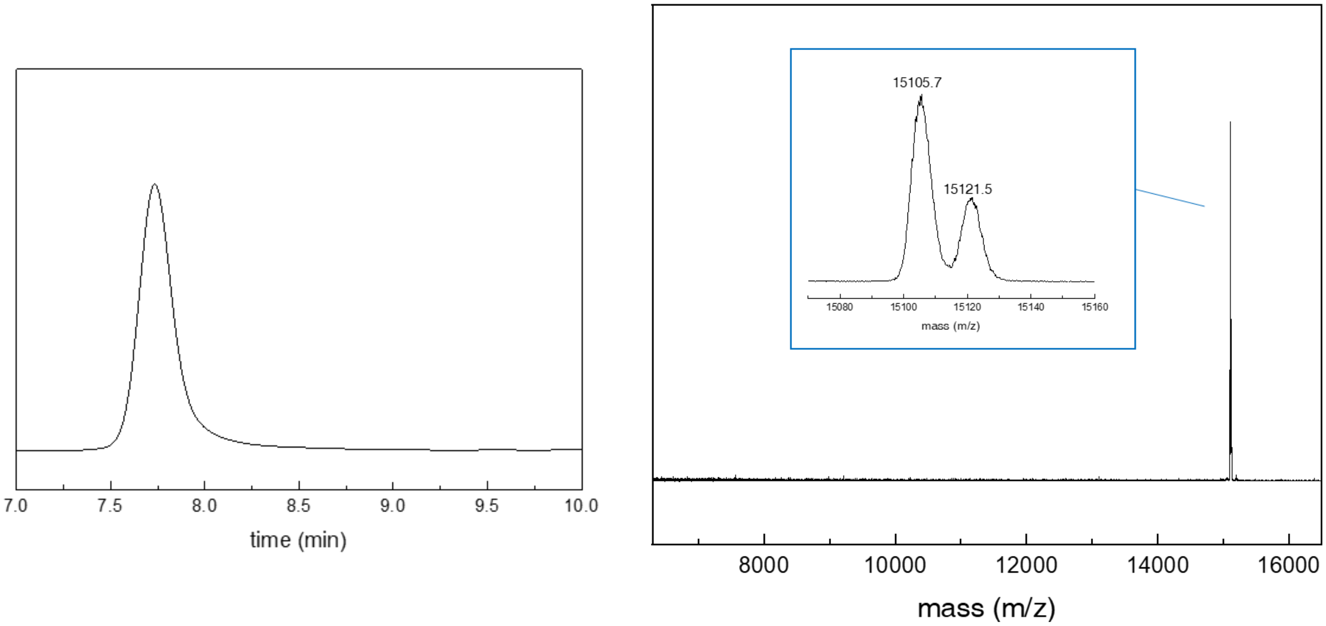

Supplementary Figure 33. GPC and MALDI-TOF MS spectra of PcL (16-byte, SEQUENCESEQUENCE) (theoretical molecular mass $[M+Na]^+$ = 15100.7 Da).

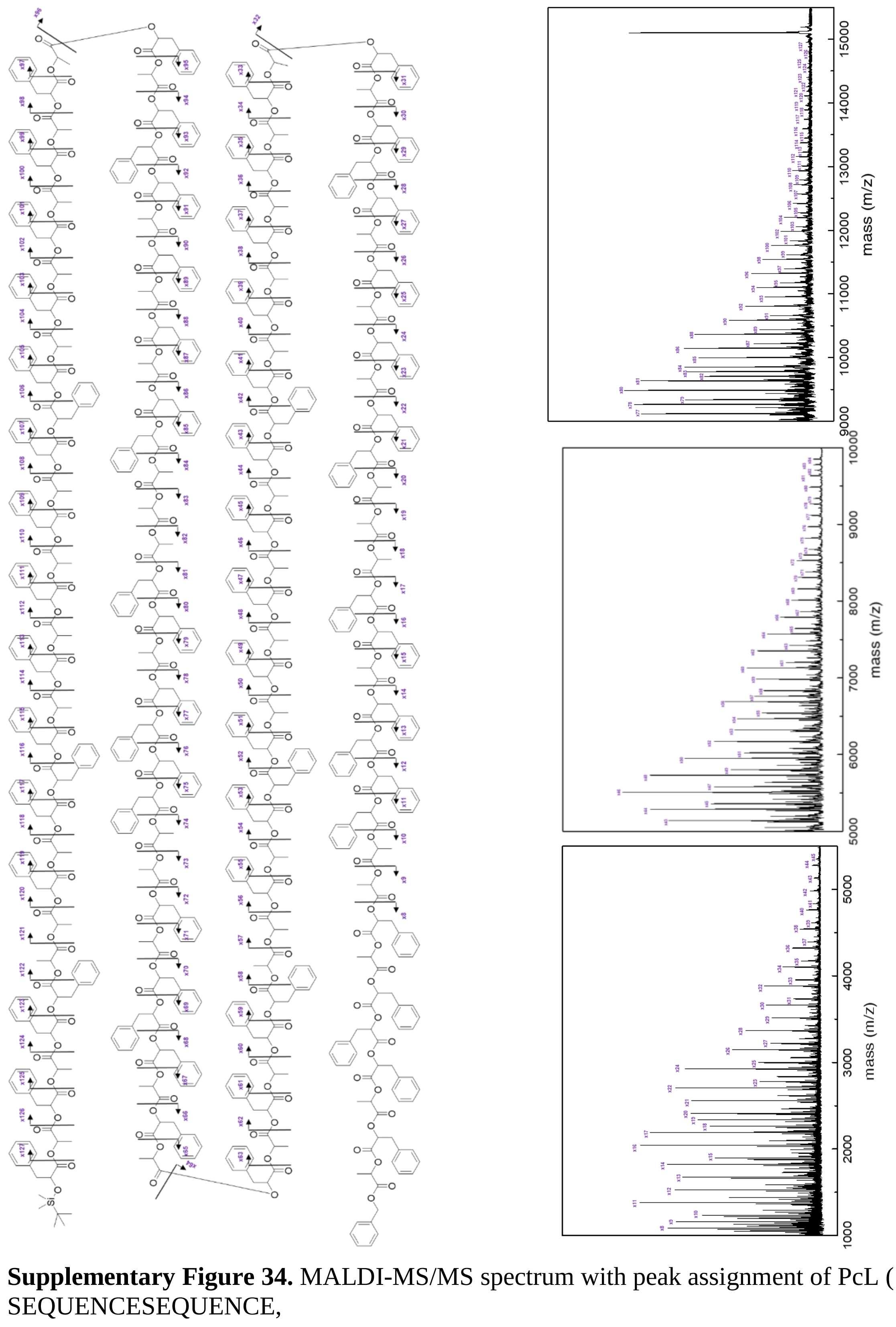

Supplementary Figure 34. MALDI-MS/MS spectrum with peak assignment of PcL (16-byte, SEQUENCESEQUENCE, PLPLPPLLPLPPPLPLPLPLPPPLPLPLPLPLPLPPPLPLPLPPLLLPPLPPPPLLPLPPPLPLPLPLPPLL PLPPPLPLPLPLPPPLPLPLPLPLPLPPPLPLPLPPLLLPPLPPPPLLPLPPPLPL)

Supplementary Table 14. Decoding table of PcL (16-byte, SEQUENCESEQUENCE)

Si → Bz	Calculated m/z	Found m/z	Difference	Sequence
$[M+Na]^+$	15101	15104		
x127	14838	14841	263	Si-P
x126	14766	14769	72	L
x125	14618	14621	148	P
x124	14546	14550	71	L
x123	14398	14402	148	P
x122	14250	14254	148	P
x121	14178	14181	73	L
x120	14106	14109	72	L
x119	13957	13962	147	P
x118	13885	13888	74	L
x117	13737	13739	149	P
x116	13589	13591	148	P
x115	13441	13442	149	P
x114	13369	13371	71	L
x113	13221	13223	148	P
x112	13149	13150	73	L
x111	13000	13003	147	P
x110	12928	12931	72	L
x109	12780	12782	149	P
x108	12708	12710	72	L
x107	12560	12562	148	P
x106	12412	12414	148	P
x105	12264	12266	148	P
x104	12192	12194	72	L
x103	12043	12045	149	P
x102	11971	11973	72	L
x101	11823	11825	148	P
x100	11751	11753	72	L
x99	11603	11605	148	P
x98	11531	11532	73	L
x97	11383	11383	149	P
x96	11311	11313	70	L

**x95**	11163	11164	149	P
**x94**	11090	11092	72	L
**x93**	10942	10942	150	P
**x92**	10794	10795	147	P
**x91**	10646	10647	148	P
**x90**	10574	10575	72	L
**x89**	10426	10428	147	P
**x88**	10354	10354	74	L
**x87**	10206	10206	148	P
**x86**	10133	10134	72	L
**x85**	9985	9986	148	P
**x84**	9837	9837	149	P
**x83**	9765	9766	71	L
**x82**	9693	9694	72	L
**x81**	9621	9622	72	L
**x80**	9473	9473	149	P
**x79**	9325	9324	149	P
**x78**	9253	9252	72	L
**x77**	9104	9104	148	P
**x76**	8956	8956	148	P
**x75**	8808	8808	148	P
**x74**	8660	8660	148	P
**x73**	8588	8588	72	L
**x72**	8516	8516	72	L
**x71**	8368	8367	149	P
**x70**	8296	8296	71	L
**x69**	8147	8147	149	P
**x68**	7999	7999	148	P
**x67**	7851	7850	149	P
**x66**	7779	7779	71	L
**x65**	7631	7630	149	P
**x64**	7559	7558	72	L
**x63**	7411	7410	148	P
**x62**	7339	7338	72	L
**x61**	7190	7190	148	P
**x60**	7118	7118	72	L
**x59**	6970	6969	149	P

**x58**	6822	6821	148	P
**x57**	6750	6749	72	L
**x56**	6678	6677	72	L
**x55**	6530	6529	148	P
**x54**	6458	6456	73	L
**x53**	6310	6308	148	P
**x52**	6161	6160	148	P
**x51**	6013	6012	148	P
**x50**	5941	5941	71	L
**x49**	5793	5792	149	P
**x48**	5721	5721	71	L
**x47**	5573	5572	149	P
**x46**	5501	5500	72	L
**x45**	5353	5352	148	P
**x44**	5280	5281	71	L
**x43**	5132	5132	149	P
**x42**	4984	4983	149	P
**x41**	4836	4836	147	P
**x40**	4764	4764	72	L
**x39**	4616	4615	149	P
**x38**	4544	4544	71	L
**x37**	4396	4395	149	P
**x36**	4323	4322	73	L
**x35**	4175	4174	148	P
**x34**	4103	4102	72	L
**x33**	3955	3954	148	P
**x32**	3883	3882	72	L
**x31**	3735	3734	148	P
**x30**	3663	3662	72	L
**x29**	3515	3514	148	P
**x28**	3366	3366	148	P
**x27**	3218	3218	148	P
**x26**	3146	3146	72	L
**x25**	2998	2998	148	P
**x24**	2926	2925	73	L
**x23**	2778	2777	148	P
**x22**	2706	2705	72	L

**x21**	2558	2557	148	P
**x20**	2409	2409	148	P
**x19**	2337	2337	72	L
**x18**	2265	2265	72	L
**x17**	2193	2193	72	L
**x16**	2045	2045	148	P
**x15**	1897	1897	148	P
**x14**	1825	1825	72	L
**x13**	1677	1677	148	P
**x12**	1529	1529	148	P
**x11**	1380	1379	150	P
**x10**	1232	1231	148	P
**x9**	1160	1159	72	L
**x8**	1088	1087	72	L

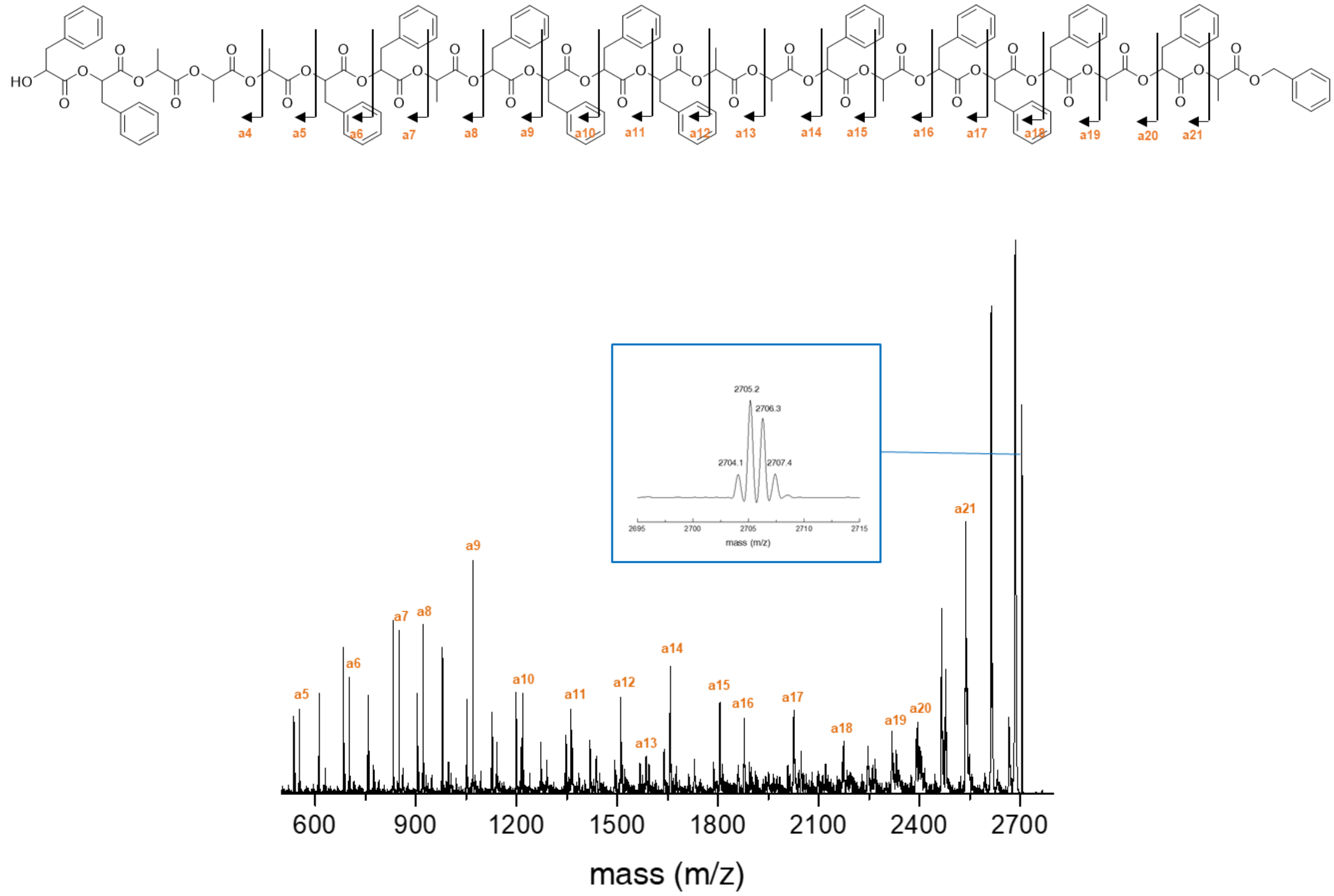

Supplementary Figure 35. MALDI-MS/MS spectrum with peak assignment of hydroloyzed fragment, x22 (theoretical isotopic maximum mass $[M + Na]^+$ = 2704.9 Da)

Supplementary Table 15. Decoding table of hydroloyzed fragment, x22 (HO-PPLLLPPLPPPPLLPLP PPLPL-Bz)

Bz → OH	Calculated m/z	Found m/z	Difference	Sequence
[M+Na]$^{+}$	2703.9	2705.2		
a21	2541.8	2543.0	162.2	Bz-L
a20	2393.8	2395.1	147.9	P
a19	2321.8	2323.0	72.1	L
a18	2173.7	2174.9	148.1	P
a17	2025.7	2026.3	148.6	P
a16	1877.6	1877.3	149.0	P
a15	1805.6	1805.5	71.8	L
a14	1657.5	1657.4	148.1	P
a13	1585.5	1585.4	72.0	L
a12	1513.5	1513.4	72.0	L
a11	1365.4	1365.4	148.0	P
a10	1217.4	1217.4	148.0	P
a9	1069.3	1069.4	148.0	P
a8	921.3	921.4	148.0	P
a7	849.3	849.4	72.0	L
a6	701.2	701.3	148.1	P
a5	553.2	553.3	148.0	P
a4	481.1	481.3	72.0	L
